# CircHIPK3 negatively regulates autophagy by blocking VCP binding to the Beclin 1 complex in bladder cancer

**DOI:** 10.1007/s12672-023-00689-0

**Published:** 2023-06-03

**Authors:** Chong Wang, Tiantian Liu, Jiawei Wang, Chao Cheng, Ze Zhang, Jingwei Zhang, Houbao Huang, Yawei Li

**Affiliations:** 1 Department of Urology, The Fifth Affiliated Hospital Sun Yat-Sen University, Zhuhai, 519000 Guangdong People’s Republic of China; 2 Department of Urology, The First Affiliated Hospital (Yijishan Hospital) of Wannan Medical College, 2 Zheshan West Road, Wuhu, 241001 People’s Republic of China; 3 School of Medicine, Yangpu Hospital, Tongji University, Shanghai, 200090 China

**Keywords:** Circular RNA, RNA-binding proteins (RBPs), Autophagy, VCP, Bladder cancer

## Abstract

Circular RNA HIPK3 (circHIPK3) mediates the progression of multiple cancers, including bladder cancer, by regulating cell migration, autophagy and epithelial mesenchymal transition. However, the mechanism by which circHIPK3 regulates autophagy in bladder cancer cells remains unclear. Autophagy is a common self-protection mechanism in eukaryotic cells and is essential for cell survival and death regulation. However, it is unclear whether circHIPK3 affects the level of autophagy in bladder cancer through binding proteins, and the potential regulatory mechanism is unknown. Here, we found that circHIPK3 levels were significantly lower and autophagy-related proteins were significantly upregulated in bladder cancer cells and tissues compared to normal controls. CircHIPK3 downregulation promoted bladder cancer cell proliferation, while circHIPK3 overexpression inhibited proliferation. CircHIPK3 overexpression significantly suppressed autophagy in bladder cancer cells. Overexpression of circHIPK3 did not affect VCP protein expression but inhibited the VCP/Beclin 1 interaction. VCP also stabilized Beclin 1 and promoted autophagy in bladder cancer cells by downregulating ataxin-3. Thus, circHIPK3 may play an important role in bladder cancer by inhibiting VCP-mediated autophagy.

**Supplementary Information:**

The online version contains supplementary material available at 10.1007/s12672-023-00689-0.

## Introduction

Bladder cancer is one of the most common malignant tumors of the urinary system worldwide and has a high mortality rate; it is ranked in the top 10 in terms of incidence among all types of cancers in China [1, 2]. Worldwide, the overall annual incidence of bladder cancer is 9.5 per 100,000 men and 2.4 per 100,000 women [3]. Although there are many clinical treatments available the incidence rate has been increasing every year [4]. The pathogenesis of bladder cancer may be related to increased proliferation and decreased apoptosis caused by the activation of oncogenes and the inactivation of tumor suppressor genes [5]. The high incidence and recurrence rates of bladder cancer make bladder cancer difficult to cure [6]. Because of the rapid development of molecular technologies, therapeutic targets and diagnostic markers have been found [7]. The discovery of these molecules will greatly facilitate the diagnosis and treatment of bladder cancer [8]. Because of their important biological functions, noncoding RNAs have gradually become the focus of medical research. Several studies have confirmed that noncoding RNAs can participate in the pathological progression of a variety of malignant tumors [9]. Initially, circular RNAs (circRNAs) were rarely reported or were misinterpreted as noise from splicing errors. With the development of RNA-seq technology and bioinformatics, several new circRNAs have been found to be explicitly involved in the pathological mechanisms of diseases, especially cancers [10, 11]. Circular RNA HIPK3 (circHIPK3) is derived from chromosomal region 11p13 and originates from the second exon of HIPK3; it has been reported in a variety of cancers, such as gastric cancer [12] and lung cancer [13]. Previously, our studies have demonstrated that circHIPK3 sponges miR-558 to suppress heparanase expression in bladder cancer cells [14]. With circRNA research, the complex regulatory network of noncoding RNAs can be elucidated, which will play an essential role in the prognostication and treatment of malignant tumors [15].

Autophagy is the self-digestion of organelles and the degradation of damaged, denatured or aged macromolecules by lysosomes under the influence of environmental factors [16]. Therefore, autophagy can be used as a self-protection mechanism in cells. Autophagy exists widely in eukaryotic cells and plays an important role in regulating cell survival and death [17]. Autophagy can be divided into three different types: macroautophagy, microautophagy, and chaperone-mediated autophagy. In autophagy research, biochemical detection of autophagosome membrane marker proteins has become a common method [18]. Microtubule-associated protein 1 light chain 3 (LC3) is a microtubule-associated protein homolog of ATG8, and LC3A, LC3B and LC3C are LC3 isoforms. The molecular weight of LC3-II is greater than that of LC3-I, but due to the extremely strong hydrophobic force between its molecules. The mammalian homolog of ATG6, BECN1, encodes a protein called Beclin1 that regulates the formation of phages. Beclin 1 is a component of the class III PI3K complex and participates in the formation of phagosomes, thus promoting autophagy [19]. Valosin-containing protein (VCP, also known as P97) is an ATPase involved in a variety of cell activities. Studies have found that defects in VCP are the cause of inclusion body myopathy with early-onset Paget disease and frontotemporal dementia [20]. In addition, VCP is closely related to autophagy, as it can promote the maturation of autophagosomes and the fusion of lysosomes. In addition, a study also found that VCP promoted the early initiation of autophagy by stabilizing the expression level of Beclin 1 [20]. However, the role of VCP in cancer is still unclear, and whether VCP can promote autophagy levels by stabilizing Beclin 1 in bladder cancer is also unclear.

In this study, we found that circHIPK3 was expressed at low levels in bladder cancer tissue and may serve as a tumor suppressor gene. The relationship between circHIPK3 and VCP was confirmed by chromatin isolation by RNA purification (CHIRP) and RNA binding protein immunoprecipitation (RIP). Coimmunoprecipitation (Co-IP) verified that overexpression of circHIPK3 could inhibit the interaction of VCP/Beclin 1. Overall, circHIPK3 could affect bladder cancer progression by inhibiting autophagy levels.

## Materials and methods

### Samples and cell lines

A total of 38 specimens of bladder cancer and adjacent tissues were obtained at The First Affiliated Hospital of Wannan Medical College. Postoperative bladder cancer and adjacent tissues were immediately frozen in liquid nitrogen and stored in a freezer at -80 °C. The study was approved by the local Ethics Committee of The First Affiliated Hospital of Wannan Medical College, and informed consent was obtained from all patients. Human bladder epithelial cells (SV-HUC-1 cells) and T24 and EJ bladder cancer cells were obtained from FuHeng BioLogy (China) and cultured in RPMI 1640 (HyClone, USA) with 10% fetal bovine serum (FBS; Gibco).

### RT‒PCR analysis

Total RNA was isolated from tissues and cells using the standard TRIzol method (TIANGEN, Beijing, China), and cDNA synthesis was performed with a High Capacity cDNA Reverse Transcription Kit (Takara, Dalian, China). RT‒PCR was performed using SYBR Premix Ex Taq TM (Tli RNaseH Plus) (Takara), and mRNA levels were normalized to those of an internal control, for instance, GAPDH. Ct values for the targets were normalized to those of GAPDH. Each sample was analyzed in triplicate. circHIPK3 si-1 sense 5'-GGUACUACAGGUAUGGCCUTT-3’; circHIPK3 si-1 antisense 5'-AGGCCAUACCUGUAGUACCGA-3'; CircHIPK3 si-2 sense 5'- UACUACAGGUAUGGCCUCATT-3'; circHIPK3 si-2 antisense 5'- UGAGGCCAUACCUGUAGUACC-3'; circHIPK3 si-3 sense 5'- UCGGUACUACAGGUAUGGCTT-3'; circHIPK3 si-3 antisense 5'- GCCAUACCUGUAGUACCGAGA-3'.

### Cell counting Kit-8 (CCK-8) assay

Cell suspensions (100 μL/well) were inoculated into 96-well plates. The plates were placed in an incubator for preculture for 24 h. After adding 10 μL of CCK-8 solution to each well (to avoid the formation of bubbles in wells, which might affect the OD reading). The plates were placed in the incubator and incubated for 1–4 h.

### Western blot analysis

SDS‒PAGE reagents, 10X electrophoresis solutions, transfer buffer solution and Tris–HCl-Tween (TBST) were prepared according to standard protocols. Antibodies against human LC3B (Proteintech Group, Rosemont, IL, USA), SQSTM1/p62 (Proteintech Group, Rosemont, IL, USA), Beclin 1 (Proteintech Group, Rosemont, IL, USA), VCP (Proteintech Group, Rosemont, IL, USA), ataxin-3 (Proteintech Group, Rosemont, IL, USA), and β-actin (Proteintech Group Rosemont IL USA) were used.

### Bioinformatics

CircNet (https://awi.cuhk.edu.cn) was used to analyze the pancancer module of circHIPK3, and its related gene ontology (GO) and Kyoto Encyclopedia of Genes and Genomes (KEGG) pathways. The interaction between circRNA and protein was predicted by catRAPID-omics2.0 (http://service.tartaglialab.com). STRING (https://cn.string-db.org) analyzed the protein–protein interactions (PPIs) between predicted binding proteins and proteins PTEN and ATG7, and then the PPI networks of binding proteins and PTEN&ATG7 were mapped using Cytoscape. The expression of VCP protein in cancer was analyzed using the UALCAN (http://ualcan.path.uab.edu/index.html) and GEPIA (http://gepia.cancer-pku.cn/index.html) databases. The interaction between circHIPK3 and VCP protein was predicted by RNA‒Protein Interaction Prediction (http://pridb.gdcb.iastate.edu/RPISeq/). The GO analysis of VCP was performed with the online software QuickGo (https://www.ebi.ac.uk).

### RIP assay

A RIP kit (GENESEED, Guangzhou) was used to verify the binding of VCP protein and circHIPK3 with RT‒PCR according to the instructions. In simple terms, T24 and EJ cells were lysed in RIP lysates and incubated with magnetic beads containing antibodies specifically recognizing VCP proteins. IgG was used as a negative control. RNA samples bound to the magnetic beads were eluted as RT templates, and the relative content of circHIPK3 in the eluent was analyzed by RT‒PCR. VCP (IP 0.5–4.0 μg).

### Immunoprecipitation (IP)

An immunoprecipitation kit (Beyotime, P2179 M, China) was used to perform IP to confirm PPIs. In simple terms, cells were lysed with lysate, and primary antibody for immunoprecipitation was added overnight at 4 °C. Protein A + G magnetic beads were added, centrifuged at 2500 rpm for 5 min and washed again. Finally, the boiled protein was used for western blot detection. The following antibodies were used: anti-VCP (IP: 0.5–4.0 μg for IP and 1:500–1:2000 for WB), anti-Beclin 1 (IP: 0.5–4.0 μg for IP and 1:500–1:2000 for WB), anti-ataxin-3 (IP: 0.5–4.0 μg for IP and 1:500–1:2000 for WB).

### Fluorescence in situ hybridization (FISH)

FISH of circHIPK3 with a CY3-labeled RNA fluorescence probe (Ribo Biotech) was performed. EJ cells grew to a fusion rate of 50–75% before treatment. After prehybridization according to FISH instructions (GenePharma), cells were hybridized with a CY3-labeled probe specific to circHIPK3 in a 37 °C incubator. The signals were examined under a microscope (Olympus Corporation).

### Immunofluorescence

The cells were washed and fixed with polymethanol at room temperature for 30 min. The penetrating agent was added and incubated for 15 min at room temperature. After blocking with BSA for 1 h, primary antibody was added at 4 °C overnight. A day later, fluorescent secondary antibodies were incubated and sealed with DAPI sealing tablets. The Anti-VCP (dilution ratio 1:10–1:100), anti-Beclin 1 (dilution ratio 1:50–1:500), goat anti-mouse IgG (dilution ratio 1:100), and goat anti-rabbit IgG (dilution ratio 1:100) antibodies were used.

### IF/FISH

After prehybridization according to FISH instructions (GenePharma), the cells were continuously incubated with the corresponding primary antibody at 4 °C overnight. After washing three times, the secondary antibody was added dropwise and incubated at room temperature.

### Transmission electron microscopy

The resolution of the transmission electron microscope (TEM) was 0.1 ~ 0.2 nm, and the images were magnified tens of thousands to millions of times, enabling observation of the ultrastructure. In this study, the cells were fixed with 2.5% glutaraldehyde for 2 h, dehydrated with different concentrations of ethanol gradient for 15 min (70% ethanol overnight), and then dehydrated with 100% ethanol 3 times for 20 min each. Acetone was applied twice, each time for 15 min, and then removed.

### Chemical reagents

DBeQ (HY-15945) was purchased from MCE (China).

### Chromatin isolation by RNA purification (CHIRP)

The specific biotin probe (Ribo Biotech) was hybridized with circRNA overnight, and Streptomyces Magnetic Beads were then added for 2–4 h. After repeated washing, the final complex was collected, and the target RNA and DNA sequences were determined by RT‒PCR or sequencing, or proteins were determined by Western blot or mass spectrometry.

### 5-Ethynyl-20-deoxyuridine (EdU) assay

A specific reaction based on EdU and Apollo fluorescent dye can be used to rapidly detect cell DNA replication activity. This assay can quickly and accurately detect the proliferation of cells. After washing with PBS 3 times, the reaction mixture was treated with 300 µL of 1 × Apollo for 30 min. After staining with 100 μL Hoechst 33,342 (5 μg/mL) for 30 min, the DNA content of the cells was observed under a fluorescence microscope (Ribo Biotech, China).

### Screening of stably transfected cell lines

Lentivirus was incubated with T24 plasmid (GenePharma, Shanghai, China) in a 96-well plate. Fresh medium was added after 24 h. After 48 h, the fluorescence intensity was measured under a microscope. When the cells reached an appropriate density, the infected cells were transplanted into 24-well plates.

### RNAi and cell transfection

To downregulate circHIPK3, three siRNAs targeting the back-splice junction of circHIPK3 (circHIPK3 si-1, circHIPK3 si-2, and circHIPK3 si-3) and si-NC were synthesized by GenePharma (Shanghai, China). Transfection was carried out using RNAi-mate (GenePharma, Shanghai, China) according to the manufacturer’s instructions.

### Animal experiments

The animal experiment was approved by the Animal Care Committee of Wannan Medical University. To investigate the role of circHIPK3 in tumor growth in vivo, BALB/c nude mice (4 weeks old, female) were randomly divided into two groups (4 mice in each group). T24 cells stably transfected with a circHIPK3 overexpression vector or empty vector were subcutaneously injected into the right axilla of nude mice (approximately 5 × 10^6^ cells per mouse). After 4 weeks, tumor tissues from mice were collected, and tumor weight, volume and gene expression were assessed.

### Statistical analysis

Data were analyzed using GraphPad Prism Software Version 7.0a (GraphPad, San Diego, CA). All the data are presented as the means ± standard errors of the means (SEMs). ANOVA was used to assess differences among more than two groups and Student's t tests were used to evaluate differences between two groups, with P < 0.05 considered to indicate significance.

## Results

### CircHIPK3 is expressed at lower expressed levels in bladder cancer cell lines and tissues than in corresponding controls and is mainly localized in the cytoplasm

**Fig. 1:**
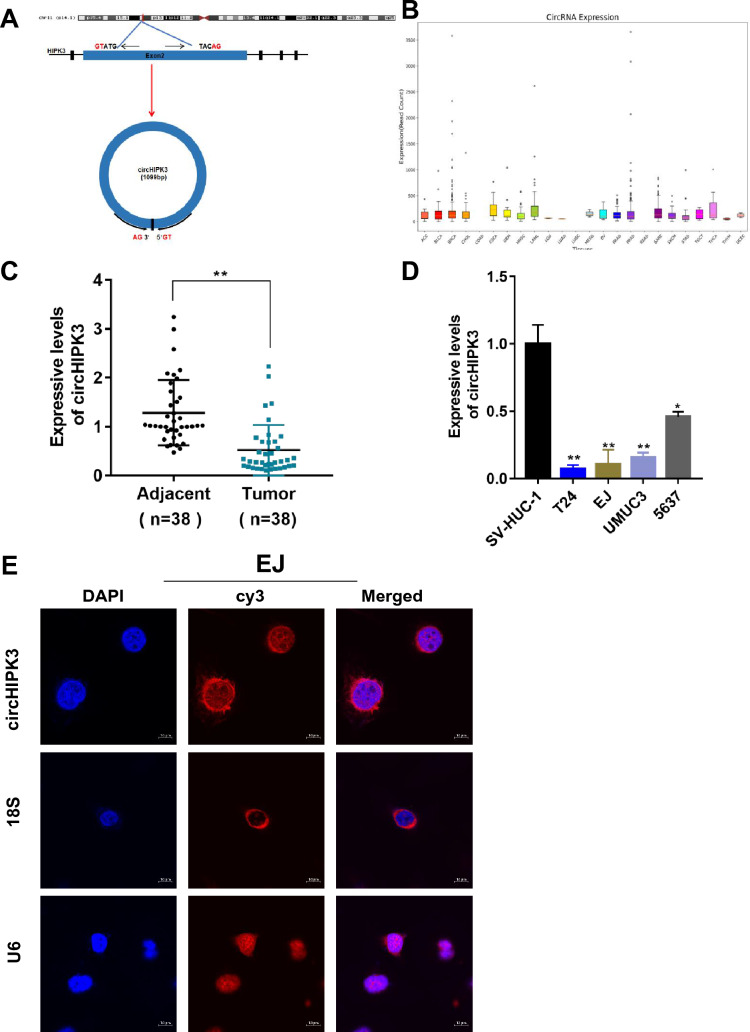
Formation, function, expression and localization of circHIPK3. **A** Schematic illustration showing the circularization of HIPK3 exon 2 forming circHIPK3 and the sequence. RT‒PCR analyses of the expression of circHIPK3 in various cell lines and tissues. **B** The CircNET database was used to analyze the expression of circHIPK3 in various tissues. **C**, **D** The expression of circHIPK3 in bladder cancer tissues and cell lines was detected by RT‒PCR. **E** A FISH assay was performed using a CY3-labeled antisense probe (circHIPK3) to detect the subcellular localization of circHIPK3 in EJ cells. U6 and 18S were used as nuclear and cytoplasmic markers, respectively. The nuclei were stained with 4, 6-diamino-2-phenylindole (DAPI), Scale, 10 microns. All data are presented as the means ± SDs of at least 3 independent experiments. * P < 0.05, ** P < 0.01

The circular RNA HIPK3 (hsa_circ_0000284, 1099 bp) is derived from the HIPK3 gene via the head-to-tail splicing of exon 2 (Fig. 1A). Database analysis showed that circHIPK3 was expressed in various human tissues (Fig. 1B). To explore the role of circHIPK3 in bladder cancer development, we first detected the expression level of circHIPK3 in 38 pairs of bladder cancer tissues and tumor-adjacent tissues. Of the 38 clinical samples, only 4 samples had high expression, and the rest had low expression (Fig. 1C). Then, we detected the expression level of circHIPK3 in different bladder cancer cell lines and SV-HUC-1 cells as a control. The data showed that circHIPK3 expression was lower in bladder cancer cell lines than in normal urothelial cells (Fig. 1D). Finally, two cell lines (T24 and EJ) were selected for the following experiments. We collected information for the 38 clinical samples, which showed that circHIPK3 was negatively correlated with bladder cancer grade, invasion, and lymph node metastasis (Table 1). To further explore the function of circHIPK3, we used FISH to determine its subcellular localization. The results showed that circHIPK3 was preferentially localized in the cytoplasm (Fig. 1E).

**Table 1:** Relationship between the expression of circHIPK3 and clinicopathological characteristics in 38 patients with bladder cancer

Parameter	Cases	Low (%)	High (%)
Sex			
Male	24	22 (92)	2 (8)
Female	14	12 (86)	2 (14)
Age at surgery			
< 60	18	16 (89)	2 (11)
> = 60	20	18 (90)	2 (10)
	Cases	pTa-T1 (%)	pT2-T4 (%)
Pathological stage			
Low	10	7(70)	3 (30)
High	28	27 (96)	1 (4)
	Cases	Low (%)	High (%)
Grade			
Low	11	8 (73)	3 (27)
High	27	26 (96)	1 (4)
	Cases	Absent (%)	Present (%)
Lymph node metastasis			
Absent	20	16 (80)	4 (20)
Present	18	18 (100)	0 (0)
	Cases	Absent (%)	Present (%)
Vascular involvement			
Absent	19	15 (79)	4 (21)
Present	19	19 (100)	0 (0)

### CircHIPK3 represses the growth of bladder cancer cells

**Fig. 2:**
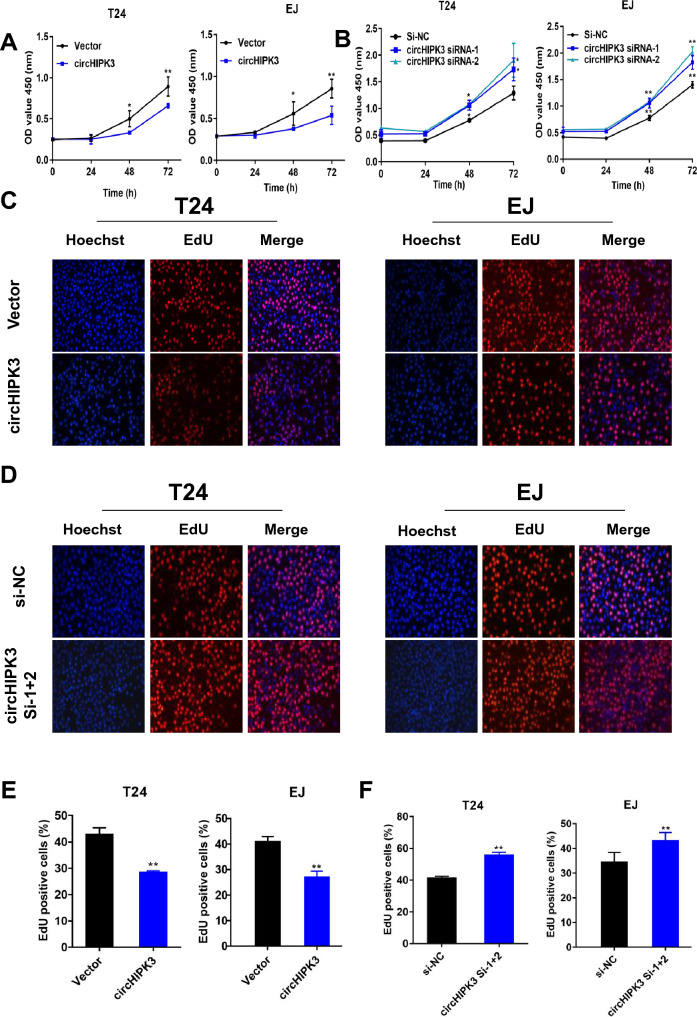
CircHIPK3 expression is negatively correlated with the proliferation of bladder cancer cell lines. **A**, **B** Once the cells were growing exponentially, siRNAs were added, and the cells were cultured for 24 h. The CCK-8 assay was used to measure cell viability. There was a significant difference after 48 h. **C**, **D** Elevated expression of circHIPK3 inhibited DNA replication in T24 and EJ cells. The EdU assay showed that the DNA replication seen with knockdown of circHIPK3 expression was increased in T24 and EJ cells compared to that seen in the control. **E**, **F** The proliferation rate was calculated and is depicted in the bar chart (*P < 0.05, **P < 0.01)

To assess the function of circHIPK3 in bladder cancer cells, we transfected T24 and EJ cells with three siRNAs (si-NC, circHIPK3 si-1, and circHIPK3 si-2) and lentivirus-produced circHIPK3 expression constructs (Supplemental Fig. 1A–D) and performed functional assays to determine cell viability and EdU incorporation. The CCK-8 experiment proved that cell proliferation was suppressed in T24 and EJ cells transfected with circHIPK3 overexpression vector compared with those transfected with control vector. On the other hand, the viability of T24 and EJ cells with circHIPK3 downregulation by circHIPK3 si-1 and circHIPK3 si-2 was significantly increased compared with that of cells transfected with si-NC (Fig. 2A, B). In addition, EdU experiments were performed in two groups. We mixed two Si-RNAs (Si-1 + 2) and transfected the cells. CircHIPK3 overexpression in T24 and EJ cell lines inhibited DNA replication and cell proliferation. Knockdown of circHIPK3 promoted DNA replication and proliferation (Fig. 2C, D). The histogram shows the corresponding proliferation rates (Fig. 2E, F).

### CircHIPK3 negatively regulates autophagy in bladder cancer cells

**Fig. 3:**
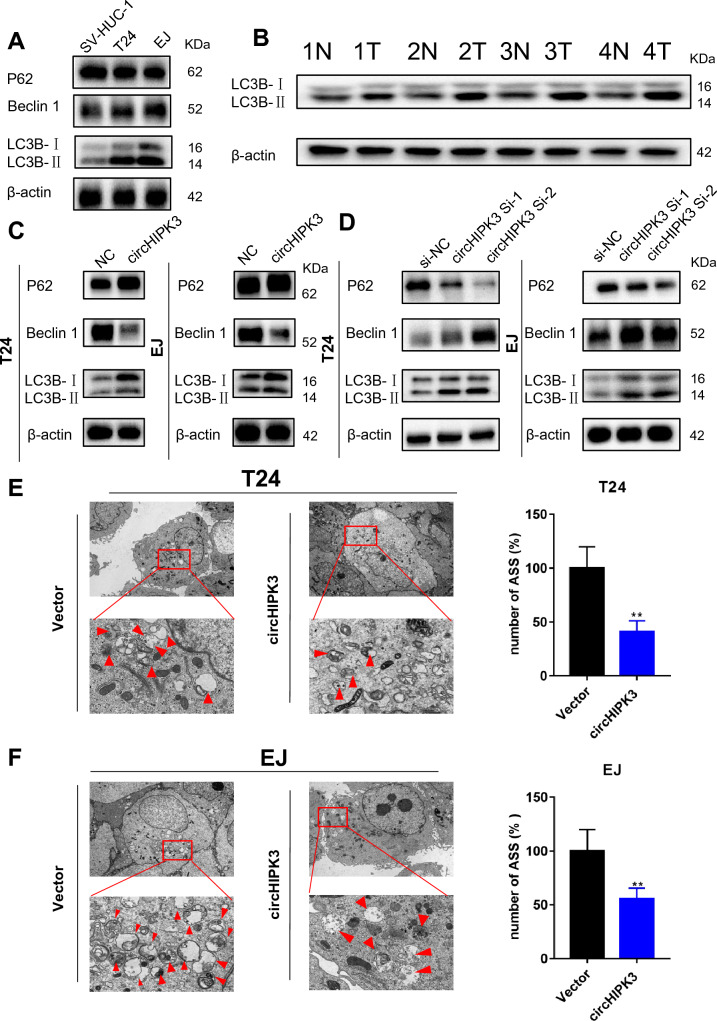
Overexpression of circHIPK3 inhibits autophagy. **A**, **B** WB was used to assess the protein expression of P62 and Beclin 1 and the conversion of LC3B-I to LC3B-II. β-actin was used as a protein loading control. **C**, **D** Transfection of the T24 and EJ cell lines with circHIPK3 si-1 and circHIPK3 si-2 increased LC3B-II accumulation. The upper LC3B band represents LC3B-I, and the lower band represents LC3B-II. **E**, **F** Representative TEM images of the intracellular ultrastructures of T24 and EJ cells. The red arrows indicate ASSs. The image on the right shows a partial enlargement of the red box on the left

To further explore the role of circHIPK3 in bladder cancer. GO analysis showed that circHIPK3 was closely related to autophagy (Supplemental Fig. 3A). To investigate the level of autophagy in bladder cancer cells, we assessed autophagy activation in T24 and EJ cells as well as bladder cancer tissues (n = 4). Western blot assays showed that the protein expression of LC3B-II in both human bladder cancer cell tissues was much higher than that observed in SV-HUC-1 cells or normal controls. The expression level of P62 was decreased, while that of Beclin 1 was increased (Fig. 3A, B). We further investigated whether overexpression of circHIPK3 could inhibit autophagy. The western blotting results showed that overexpression of circHIPK3 decreased the ratio of LC3B-II to LC3B-I and decreased Beclin1 expression but increased p62 expression in T24 and EJ cells (Fig. 3C). Inhibition of circHIPK3 expression yielded the opposite results (Fig. 3D). To assess changes in autophagy more intuitively. Autophagolysosomes (ASSs) in T24 and EJ cells were studied by transmission electron microscopy (TEM). ASS formation in T24 and EJ cells was decreased after circHIPK3 overexpression versus vector transfection (Fig. 3E, F).

### CircHIPK3 regulates autophagy by binding the VCP protein

**Fig. 4:**
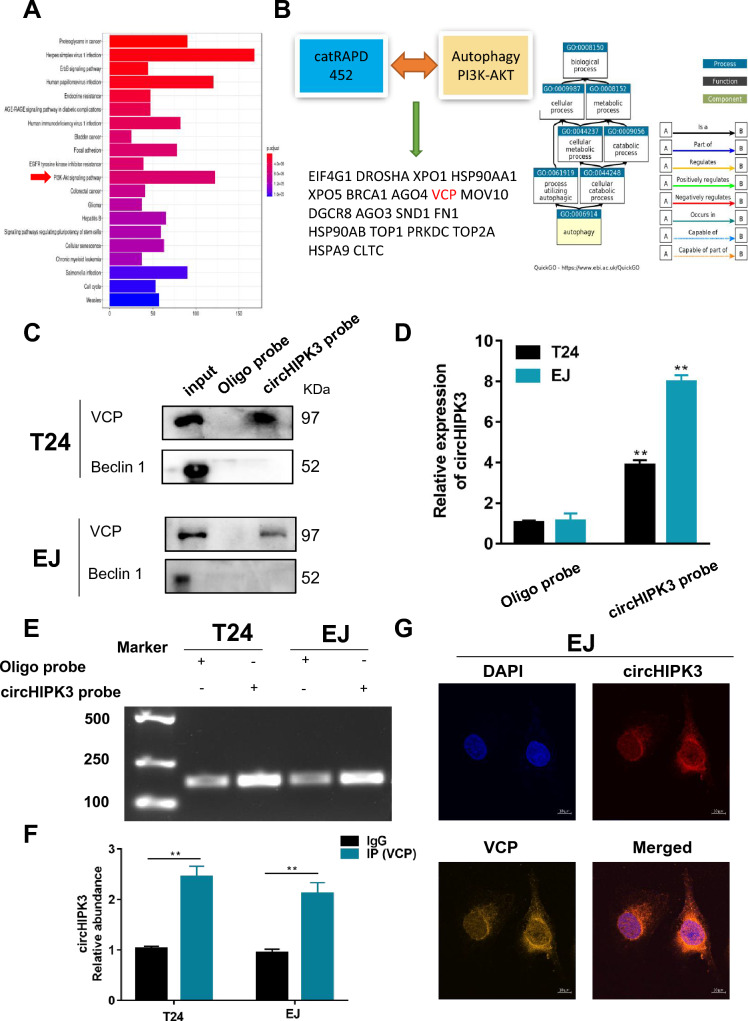
CircHIPK3 functions by sponging VCP. **A** KEGG analysis showed that circHIPK3 was closely related to the PI3K-AKT pathway. **B** RIP database analysis, autophagy-related genes and PI3K-AKT pathway-related genes predicted that they might bind to circHIPK3. GO analyzes VCP-related functions. **C** CHIRP and WB were used to analyze the interaction between VCP and circHIPK3 labeled with a probe in T24 and EJ cells. Oligo probe: antisense RNA probe, IP: immunoprecipitation. **D**, **E** Analyses of agarose gel electrophoresis and RT‒PCR confirmed the specificity of the amplification. **F** RIP and RT‒PCR assays were performed using VCP antibody directly to verify direct interaction with circHIPK3. **G** The colocalization of circHIPK3 (red) and VCP (yellow) in EJ cells was detected by RNA FISH assay combined with immunofluorescence

Studies have shown that circRNAs regulate downstream target genes mainly by competing with miRNAs and RBPs. We first predicted circHIPK3 binding proteins using a database and then reserved the (interaction propensity > 50) protein relationships for subsequent analysis (Supplemental Fig. 2A). To narrow the scope, we used the database to analyze the KEGG pathway function of circHIPK3 and found that it was closely related to autophagy-related pathways (PI3K-AKT) (Fig. 4A). The upstream target of this pathway is PTEN. Previous studies have found that ATG7 not only promotes autophagy but is also associated with the progression of bladder cancer. Therefore, we next used the previously identified proteins to predict PPIs involving the PTEN and ATG7 proteins (Supplemental Fig. 2A). We screened 19 proteins that may bind to circHIPK3. By scoring the 19 proteins in the RNA‒Protein Interaction Prediction database and GO analysis, we finally identified the interacting protein VCP (Fig. 4B and Supplemental Fig. 2B). Through probe-labeled circHIPK3 CHIRP and RIP experimental analysis, we further demonstrated that circHIPK3 could interact with VCP instead of Beclin 1 (Fig. 4C–F). Finally, RNA FISH and IF showed that circHIPK3 and VCP were predominantly colocalized in the cytoplasm (Fig. 4G).

### VCP promotes autophagy and stabilizes Beclin 1 in bladder cancer cells

**Fig. 5:**
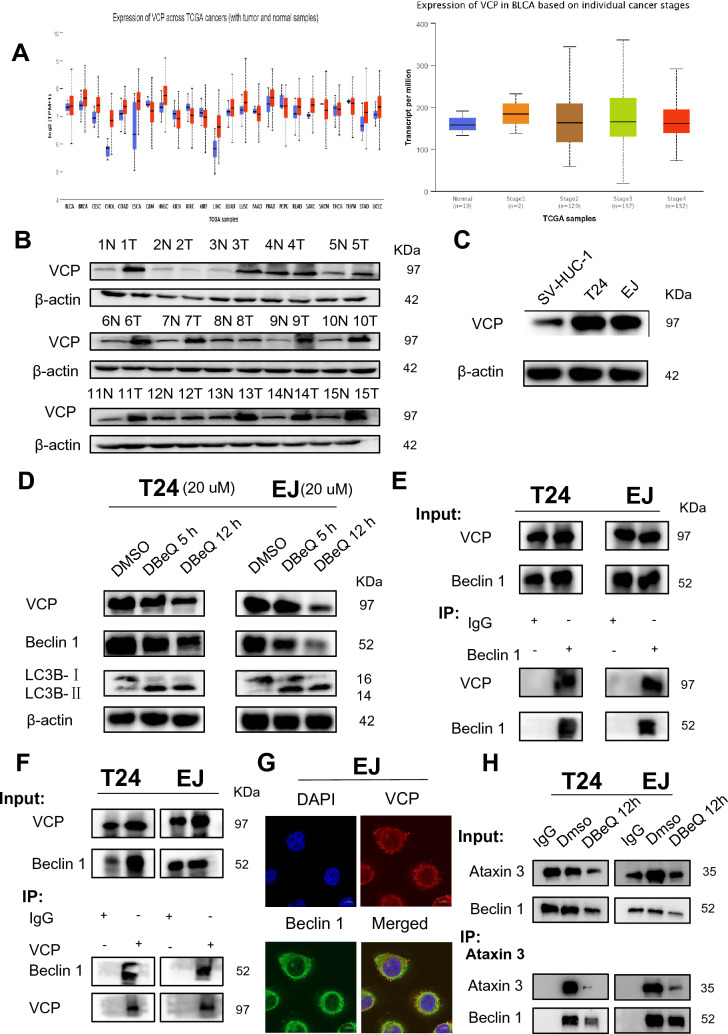
VCP stabilizes Beclin 1 via ataxin-3 to promote autophagy in bladder cancer. **A** The expression of VCP in various cancer tissues was analyzed by database. The expression of VCP was high in bladder cancer tissues and correlated with T3 and T4 (normal vs. T3 P = 0.009, normal vs. T4 P = 0.006). **B** Western blot analysis of VCP protein expression in 15 pairs of human bladder cancer and normal adjacent tissues. **C** Western blot analysis of VCP protein expression in T24, EJ and SV-HUC-1-cell lines. **D** Protein levels of VCP, Beclin 1 and LC3B-II in T24 and EJ cells treated with DBeQ (20 μM) for different times. **E**, **F** VCP interacts with Beclin 1 in T24 and EJ cells, IP: VCP, IP: Beclin 1. **G** Immunofluorescence staining was used to detect the expression of VCP (red) and Beclin 1 (green) in EJ cells. Scale bars = 10 μm. All experiments were performed in triplicate. **H** T24 and EJ cells were treated with DBeQ (20 μM, 12 h) for the IP of ataxin-3

Previous studies have shown that VCP can stabilize Beclin 1 in neurological diseases and promote the progression of autophagy [20]. We speculated that VCP may also rely on this pathway to regulate autophagy in bladder cancer. Online database analysis showed that VCP was highly expressed in most cancer tissues. Moreover, the expression of VCP increased persistently from T3 to T4 (Fig. 5A). The WB results showed that the expression of VCP in bladder cancer tissues was significantly higher than that in normal tissues (n = 15) (Fig. 5B). The expression of VCP in T24 and EJ cells was also significantly higher than that in SV-HUC-1 cells (Fig. 5C). This suggested that VCP may play the role of an oncogene in bladder cancer. Subsequently, we used an inhibitor (DBeQ) to acutely inhibit VCP activity, and the WB results showed a decrease in Beclin 1 expression and an increase in total LC3B II. This is consistent with previous studies (Fig. 5D). Then, we verified the endogenous interaction of these two proteins in bladder cancer cells, where VCP coprecipitated with Beclin 1 (Fig. 5E, F). Immunofluorescence assays confirmed that VCP and Beclin 1 colocalized in the cytoplasm (Fig. 5G, and Supplemental Fig. 3B). We also found that the inhibition of VCP reduced the levels of ataxin-3 and Beclin-1 and the ability of the two proteins interact (Fig. 5H).

### CircHIPK3 inhibits autophagy by inhibiting the interaction of the VCP and Beclin 1 proteins

**Fig. 6:**
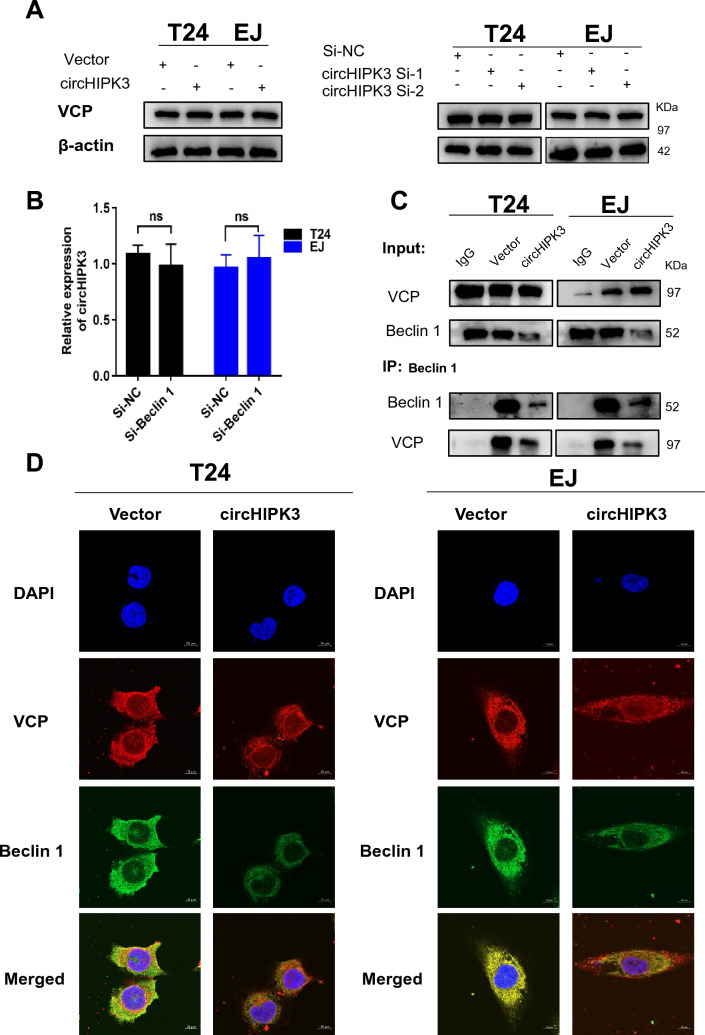
CircHIPK3/VCP/Beclin 1 axis in T24 and EJ cells. **A** Relative expression of VCP mRNA and protein in T24 and EJ cells after circHIPK3 overexpression or vector transfection. **B** Relative expression of circHIPK3 in T24 and EJ cells after si-Beclin 1 or si-NC treatment. **C** Co-IP and WB were used to analyze the influence of circHIPK3 overexpression on the interaction between VCP and Beclin 1. **D** Immunofluorescence staining was used to detect the expression of VCP (red) and Beclin 1 (green)

We wanted to verify whether circHIPK3 could inhibit the level of autophagy inhibited by VCP-stabilized Beclin 1 interactions. The WB results showed that overexpression and inhibition of circHIPK3 did not affect the changes in VCP protein levels (Fig. 6A). Similarly, knocking down Beclin 1 had no effect on the relative expression of circHIPK3 (Fig. 6B). It was implied that the circHIPK3-VCP-Beclin 1 axis might not involve positive and negative feedback. Co-IP confirmed that overexpression of circHIPK3 weakened the interaction of VCP and Beclin 1 in bladder cancer cells (Fig. 6C). Immunofluorescence staining of VCP and Beclin 1 in circHIPK3-overexpressing cells showed that circHIPK3 overexpression significantly inhibited the expression of Beclin 1, and the binding force between VCP and Beclin 1 was significantly weakened (Fig. 6D). Our results suggested that circHIPK3 could inhibit the occurrence of autophagy by interacting with VCP.

### CircHIPK3 can inhibit the effect of VCP in bladder cancer

**Fig. 7:**
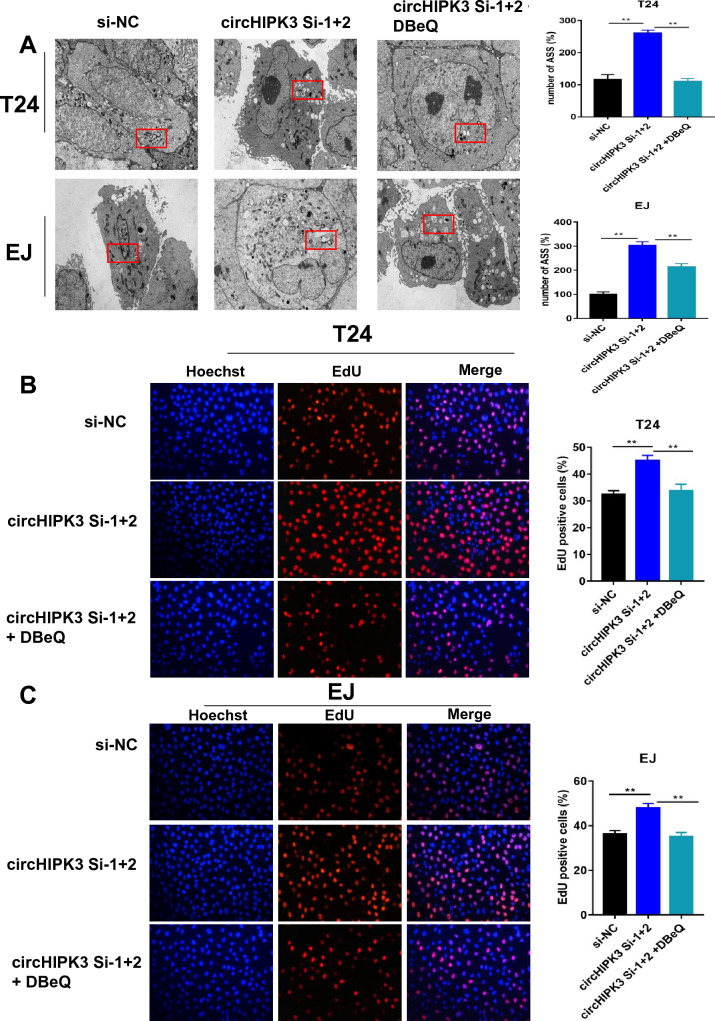
Both circHIPK3 and autophagy play important roles in regulating autophagy. **A** T24 and EJ cells were divided into three groups: si-NC, circHIPK3 si-1 + 2 and circHIPK3 si-1 + 2 + DBeQ. Representative TEM images of the intracellular ultrastructures of T24 and EJ cells. Data are the mean ± SEM, n = 3. **P < 0.01 (Student’s t test). Scale bar, 5 µm. **B**, **C** The effect of circHIPK3 and VCP on cell proliferation capability was evaluated by EdU assay in T24 and EJ cells. All results are presented as the mean ± SD of three independent experiments

In previous studies, autophagy was identified as an essential contributor to the highly aggressive nature of bladder cancer. Therefore, we further explored the role of circHIPK3 and autophagy in bladder cancer cell proliferation. The TEM results showed that the formation of autophagosomes was significantly promoted by transfection of circHIPK3 si-1 + 2 versus si-NC. However, the addition of DBeQ reversed strong induction of autophagy (Fig. 7A and Supplemental Fig. 3C). These results indicated that VCP is of vital importance in circHIPK3-mediated autophagy. In addition, EdU assay results showed that circHIPK3 si-1 + 2 significantly promoted the proliferation of bladder cancer cells compared with that in the si-NC group, but this phenomenon was reversed by the addition of DBeQ (Fig. 7B, C). Our results demonstrated that circHIPK3 could inhibit the proliferation of bladder cancer cells in vitro via VCP.

### CircHIPK3 overexpression suppresses the autophagy and proliferation of bladder cancer cells in vivo

**Fig. 8:**
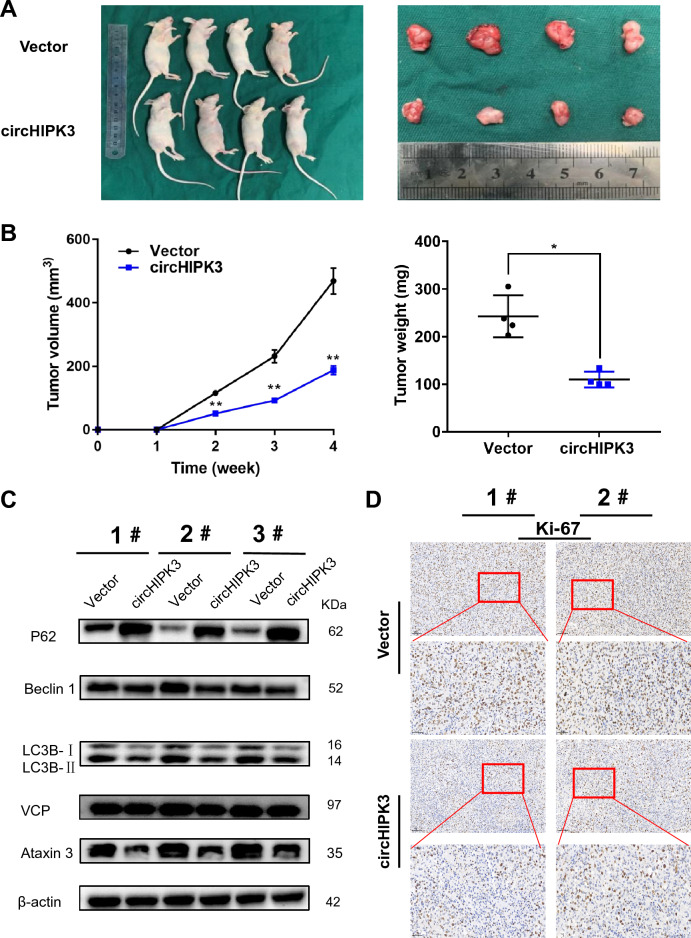
Overexpression of circHIPK3 suppresses autophagy and bladder cancer growth in vivo. **A**, **B** Subcutaneous xenografted tumors were established in BALB/c nude mice by injection of T24 cells stably transfected with circHIPK3 or control vectors. Tumor volume and weight were significantly reduced the group of nude mice that received cells transfected with circHIPK3 compared with the vector group. **C** The expression levels of P62, Beclin 1, LC3B, VCP, ataxin-3 and β-actin were detected by WB assay. **D** Immunohistochemical staining showed that circHIPK3 overexpression resulted in decreased expression of Ki-67 in tumors. Data are presented as the mean ± SEM. **P < 0.01. (Student’s t test). Scale bars, 50 and 100 μM

To confirm the role of circHIPK3 in tumor growth and autophagy in vivo, T24 cells were stably transfected with circHIPK3 and were subcutaneously injected into BALB/c nude mice. Compared with the control group, the group transfected with circHIPK3 vectors showed a reduced growth rate and weight of the transplanted tumors (Fig. 8A, B and Supplemental Fig. 4A). Next, we extracted protein from three pairs of tumor tissues. Western blotting experiments showed that the results for the levels of P62, Beclin 1, LC3B-II and LC3B-I were consistent with the results of previous in vitro studies. However, the protein levels of VCP did not change (Fig. 8C). In addition, the IHC results showed that circHIPK3 could inhibit the expression of Ki-67 (Fig. 8D and Supplemental Fig. 3D).

## Discussion

Abnormal and uncontrolled cell proliferation is a hallmark of cancer and is caused by the misregulation of gene expression [21]. CircHIPK3 is formed by back-splicing and cyclization of the second exon of the linear RNA HIPK3, which is mainly distributed in the cytoplasm. CircHIPK3 has been shown to inhibit the migration and invasion of bladder cancer cells, but the effect of circHIPK3 on the autophagy of bladder cancer cells has not been reported [14]. In this study, we found that the expression of circHIPK3 was significantly downregulated in both bladder cancer tissues and cell lines. CCK-8 and EdU experiments suggested that overexpression of circHIPK3 can inhibit the proliferation of T24 and EJ cells and that silencing of circHIPK3 can promote the proliferation of bladder cancer cells. Recent studies suggest that circHIPK3 may act as a tumor suppressor in bladder cancer and may be a potential therapeutic target.

Although autophagy is believed to be a mechanism of cell self-protection, dysregulation of autophagy-related genes can also cause cell death [22]. Studies have shown that autophagy dysregulation can lead to major diseases such as cancer, diabetes, neurodegeneration, and immune disorders [17]. In this study, we found that circHIPK3 could block the binding of the VCP protein to Beclin 1, which is contained in the PI3K complex to promote autophagy. Unlike previous studies, circHIPK3 acts by directly interacting with VCP to induce deubiquitination and inhibit its interaction with Beclin 1. Our data indicate that circHIPK3 can affect cellular proliferation and function by directly regulating autophagy, providing a novel mechanism of circRNAs in cancer progression.

Valosin-containing protein (VCP/p97) was first discovered in 1982 [23]. VCP is mainly located in the cytoplasm and belongs to the type II AAA + ATPase family [23]. VCP has many functions and is involved in protein metabolism and cell homeostasis [24]. Studies have found that VCP is involved in the initiation of autophagy or autophagosome maturation [20]. However, there are few studies on the role of VCP in cancer, especially in bladder cancer. Our bioinformatics analysis and experimental data showed that the VCP protein level was significantly higher in bladder cancer tissues than in normal tissues. VCP inhibitors could effectively inhibit the expression of VCP, and the proliferation of bladder cancer cells and autophagy levels were also affected. In terms of mechanism, previous studies have found that VCP and Beclin 1 could interact to promote levels of autophagy [20], and our experimental results confirmed this. The Beclin-1-Vps15-Vps34 complex plays an important role in the initiation of autophagy [25]. Beclin-1 not only acts as a key autophagic regulator and its specific interactor but also represents a potential therapeutic target for cancer [26]. In the future, it is necessary to find new treatments for cancer and other diseases by developing inhibitors such as those targeting VCP.

Ataxin-3 is a protein involved in various cellular processes, such as deubiquitination, cytoskeletal organization, and transcriptional regulation. According to most literature reports, the abnormal expression of the ataxin-3 gene is mostly caused by spinocerebellar ataxia type 3 (SCA3) [27]. However, the role of ataxin-3 in bladder cancer remains unclear. Previous studies have reported that VCP and ataxin-3 are related and that both can promote autophagy [23]. Our data also demonstrate a correlation between these two genes in bladder cancer. Therefore, it is important to study the initiation, progression, molecular mechanism and regulatory mechanism of autophagy. The regulatory relationship of circHIPK3/VCP/Beclin 1 deserves further comprehensive study.

With the deepening of circular RNA research, plasmid-mediated overexpression of circular RNA is unable to meet the clinical translational needs of basic research. Conducting relevant research through in vitro synthesized circular RNA is the future development trend in the field of basic circular RNA research. The results of this study revealed a new and potentially transformative method for treating doxorubicin-induced heart failure based on Circ-INSR [28]. In vitro preparation of circular RNA technology was first applied to basic scientific research, accelerating the transformation and application of basic circular RNA research. The artificial preparation technology of circular RNA will not only shine in the industrial field but also in the field of basic scientific research. In addition, there is research that establishes a new circRNA vaccine platform by encapsulating circRNA encoding antigens in lipid nanoparticles (LNP) [29], allowing circRNA to be expressed in vivo. In this platform, circRNA vaccines can activate innate/adaptive immune responses and exhibit excellent anti-tumor effects in various mouse tumor models. These studies are inspiring us to one day use circHIPK3 as a diagnostic or even therapeutic tool for bladder cancer.

## Conclusion

Overall, our results showed that the circHIPK3/VCP/Beclin 1 axis plays an important role in regulating the proliferation and autophagy of bladder cancer cells. Moreover, different from previous studies, we found for the first time that circHIPK3 can regulate protein-protein complexes by binding the protein VCP rather than through the classical ceRNA (competing endogenous RNA) pathway. In conclusion, circHIPK3 can serve as a highly specific and sensitive biomarker for bladder cancer, aiding in early detection and monitoring of treatment response.

## Supplementary Information

**Additional file 1: Figure S1.** Development of overexpression and knockdown tools for circHIPK3. (A) Cells were transfected with circHIPK3 si-1, circHIPK3 si-2, circHIPK3 si-3, and control siRNA (si-NC). (B) RT PCR was used to detect whether the expression of linear HIPK3 was affected after circHIPK3 inhibition. (C) CircHIPK3 overexpression lentivirus was stably transfected into cells. (D) RT PCR was used to detect whether the expression of linear HIPK3 was affected after circHIPK3 overexpression. Data are the mean ± SEM, n = 3. **P < 0.01 (Student’s t test). **Figure S2** Analysis of circHIPK3 Binding Proteins in online databases and binding score with VCP Protein. (A) The proteins that circHIPK3 may bind were analyzed by an online database. (B) Interaction probabilities between circHIPK3 and VCP. **Figure S3.** The biological functions of circHIPK3 and VCP, as well as the co-localization of VCP and Beclin 1 protein. (A) GO analysis of circHIPK3 revealed that it was closely related to autophagy. (B) Immunofluorescence staining was used to detect the expression of VCP (red) and Beclin 1 (green) in T24 cells. Scale bars = 10 *μ*m. All experiments were performed in triplicate. (C) The image shows a partial enlargement of the red box. The red arrows indicate ASSs. (D) Immunohistochemical staining showed that circHIPK3 overexpression resulted in decreased expression of Ki-67 in tumors. **Figure S4.** Volume and weight of the transplant tumors. (A) The Tables 1 and 2 are 4-week growth data for mouse transplanted tumors.

**Additional file 2**. Western Blotting

## Data Availability

The datasets generated during and/or analysed during the current study are available from the corresponding author on reasonable request.
