# Supplementary material for: CircHIPK3 negatively regulates autophagy by blocking VCP binding to the Beclin 1 complex in bladder cancer
Source: Discov Oncol. 2023 Jun 3;14:86. doi: 10.1007/s12672-023-00689-0 (PMC10239413; doi:10.1007/s12672-023-00689-0)
Supplement: Supplementary file 1 — Additional file 1: Figure S1. Development of overexpression and knockdown tools for circHIPK3. (A) Cells were transfected with circHIPK3 si-1, circHIPK3 si-2, circHIPK3 si-3, and control siRNA (si-NC). (B) RT PCR was used to detect whether the expression of linear HIPK3 was affected after circHIPK3 inhibition. (C) CircHIPK3 overexpression lentivirus was stably transfected into cells. (D) RT PCR was used to detect whether the expression of linear HIPK3 was affected after circHIPK3 overexpression. Data are the mean ± SEM, n = 3. **P < 0.01 (Student’s t test). Figure S2 Analysis of circHIPK3 Binding Proteins in online databases and binding score with VCP Protein. (A) The proteins that circHIPK3 may bind were analyzed by an online database. (B) Interaction probabilities between circHIPK3 and VCP. Figure S3. The biological functions of circHIPK3 and VCP, as well as the co-localization of VCP and Beclin 1 protein. (A) GO analysis of circHIPK3 revealed that it was closely related to autophagy. (B) Immunofluorescence staining was used to detect the expression of VCP (red) and Beclin 1 (green) in T24 cells. Scale bars = 10 μm. All experiments were performed in triplicate. (C) The image shows a partial enlargement of the red box. The red arrows indicate ASSs. (D) Immunohistochemical staining showed that circHIPK3 overexpression resulted in decreased expression of Ki-67 in tumors. Figure S4. Volume and weight of the transplant tumors. (A) The Tables 1 and 2 are 4-week growth data for mouse transplanted tumors. [file 12672_2023_689_MOESM1_ESM.pdf]

Supplemental Figure 1

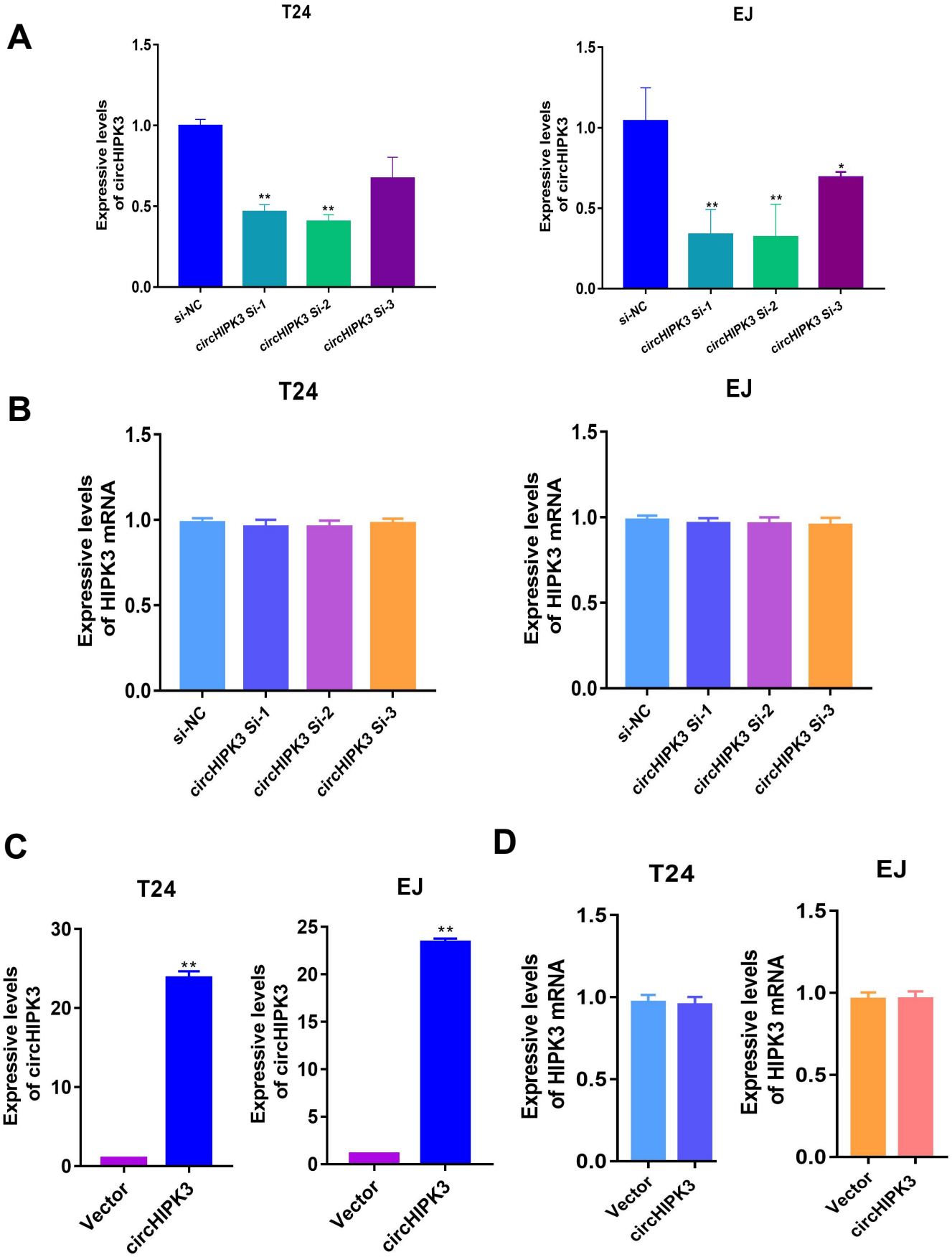

Supplemental Figure 2

A

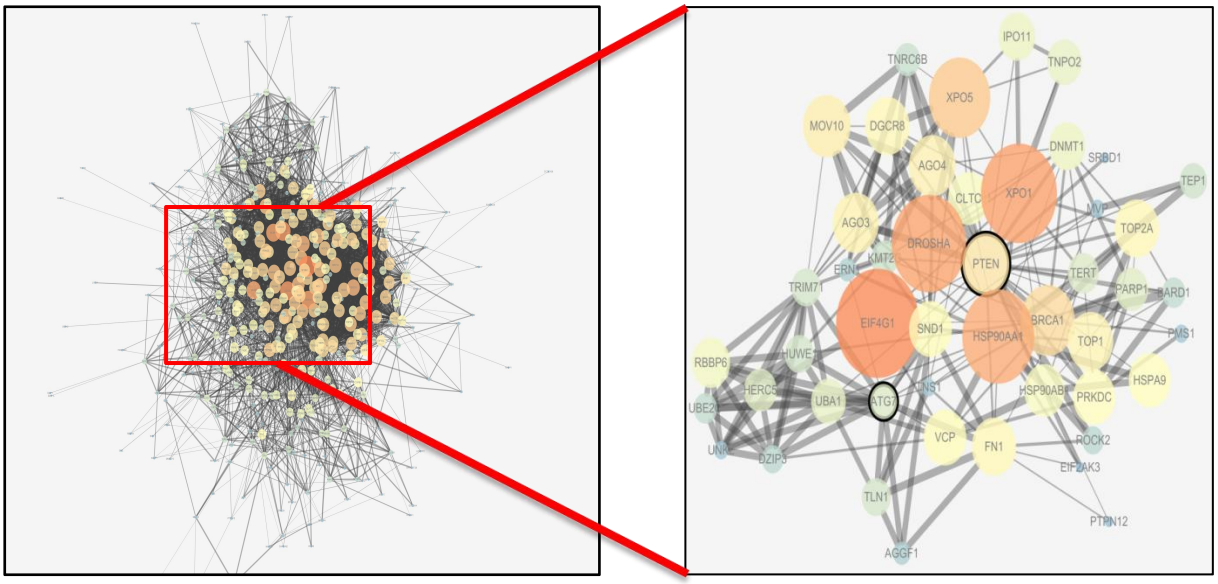

B

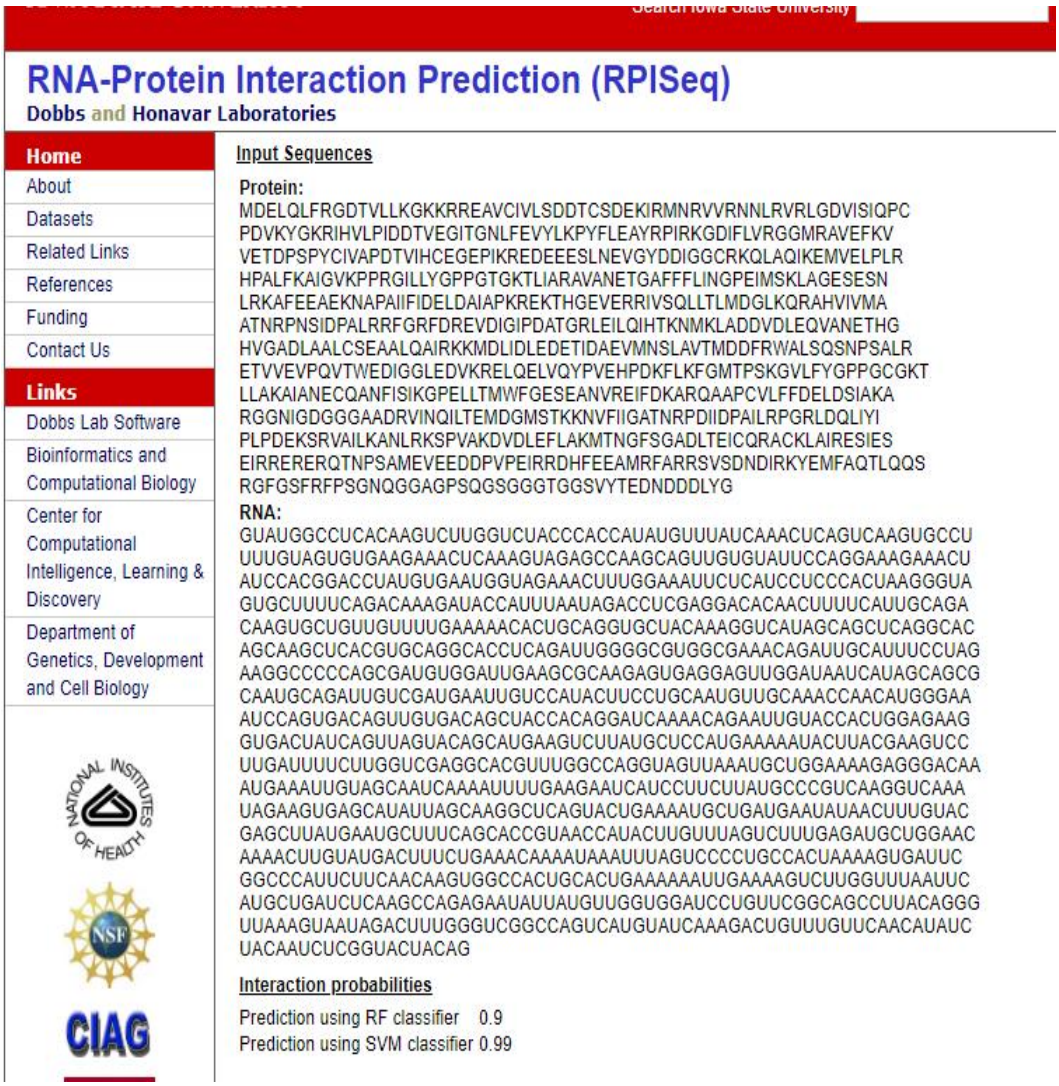

RNA-Protein Interaction Prediction (RPISeq)

Dobbs and Honavar Laboratories

Home
About
Datasets
Related Links
References
Funding
Contact Us

Links
Dobbs Lab Software
Bioinformatics and Computational Biology
Center for Computational Intelligence, Learning & Discovery
Department of Genetics, Development and Cell Biology

NATIONAL INSTITUTES OF HEALTH
NSF
CIAG

Input Sequences

Protein:
MDELQLFRGDTVLLKGKKRREAVCIVLSDDTCSDEKIRMNRVVRNNLRVRLGDVISIQPC
PDVKYGKRIHVLPIDDTVEGITGNLFEVYLKPYFLEAYRPIRKGDIFLVRGGMRAVEFKV
VETDPSPYCIVAPDTVIHCEGEPIKREDEEESLNEVGYDDIGGCRKQLAQIKEMVELPLR
HPALFKAIGVKPPRGILLYGPPGTGKTLIARAVANETGAFFFLINGPEIMSKLAGESESN
LRKAFEEAEKNAPAIIFIDELDAIAPKREKTHGEVERRIVSQLLTLMDGLKQRAHVIVMA
ATNRPNSIDPALRRFGRFDREVDIGIPDATGRLEILQIHTKNMKLADDVDLEQVANETHG
HVGADLAALCSEAALQAIRKKMDLIDLEDETIDAEVMNSLAVTMDDFRWALSQSNPSALR
ETVVEVPQVTWEDIGGLEDVKRELQELVQYPVEHPDKFLKFGMTPSKGVLFYGPPGCGKT
LLAKAIANECQANFISIKGPELLTMWFGESEANVREIFDKARQAAPCVLFFDELDSIAKA
RGGNIGDGGGAADRVINQILTEMDGMSTKKNVFIIGATNRPDIIDPAILRPGRLDQLIYI
PLPDEKSRVAILKANLRKSPVAKDVDLEFLAKMTNGFSGADLTEICQRACKLAIRESIES
EIRRERERQTNPSAMEVEEDDPVPEIRRDHFEEAMRFARRSVSDNDIRKYEMFAQTLQQS
RGFGSFRFPSGNQGGAGPSQGSGGGTGGSVYTEDNDDDLYG

RNA:
GUAUGGCCUCACAAGUCUUGGUCUACCCACCAUAUGUUUAUCAAACUCAGUCAAGUGCCU
UUUGUAGUGUGAAGAAACUCAAAGUAGAGCCAAGCAGUUGUGUAUUCCAGGAAAGAAACU
AUCCACGGACCUAUGUGAAUGGUAGAAACUUUGGAAAUUCUCAUCCUCCCACUAAGGGUA
GUGCUUUUCAGACAAAGAUACCAUUUAAUAGACCUCGAGGACACAACUUUUCAUUGCAGA
CAAGUGCUGUUGUUUUGAAAAACACUGCAGGUGCUACAAAGGUCAUAGCAGCUCAGGCAC
AGCAAGCUCACGUGCAGGCACCUCAGAUUGGGGCGUGGCGAAACAGAUUGCAUUUCCUAG
AAGGCCCCAGCGAUGUGGAUUGAAGCGCAAGAGUGAGGAGUUGGAUAAUCAUAGCAGCG
CAAUGCAGAUUGUCGAUGAAUUGUCCAUACUUCCUGCAAUGUUGCAAACCAACAUGGGAA
AUCCAGUGACAGUUGUGACAGCUACCACAGGAUCAAAACAGAAUUGUACCACUGGAGAAG
GUGACUAUCAGUUAGUACAGCAUGAAGUCUUAUGCUCCAUGAAAAAUACUUACGAAGUCC
UUGAUUUUCUUGGUCGAGGCACGUUUGGCCAGGUAGUUAAAUGCUGGAAAAGAGGGACAA
AUGAAAUUGUAGCAAUCAAAAUUUUGAAGAAUCAUCCUUCUUAUGCCCGUCAAGGUCAAA
UAGAAGUGAGCAUAUUAGCAAGGCUCAGUACUGAAAAUGCUGAUGAAUAUAACUUUGUAC
GAGCUUAUGAAUGCUUUCAGCACCGUAACCAUACUUGUUUAGUCUUUGAGAUGCUGGAAC
AAAACUUGUAUGACUUUCUGAAACAAAAUAAAUUUAGUCCCCUGCCACUAAAAGUGAUUC
GGCCCAUUCUUCAACAAGUGGCCACUGCACUGAAAAAAUUGAAAAGUCUUGGUUUAAUUC
AUGCUGAUCUCAAGCCAGAGAAUAUUAUGUUGGUGGAUCCUGUUCGGCAGCCUUACAGGG
UUAAAGUAAUAGACUUUGGGUCGGCCAGUCAUGUAUCAAAGACUGUUUGUUCAACAUAUC
UACAAUCUCGGUACUACAG

Interaction probabilities

Prediction using RF classifier 0.9
Prediction using SVM classifier 0.99

Supplemental Figure 3

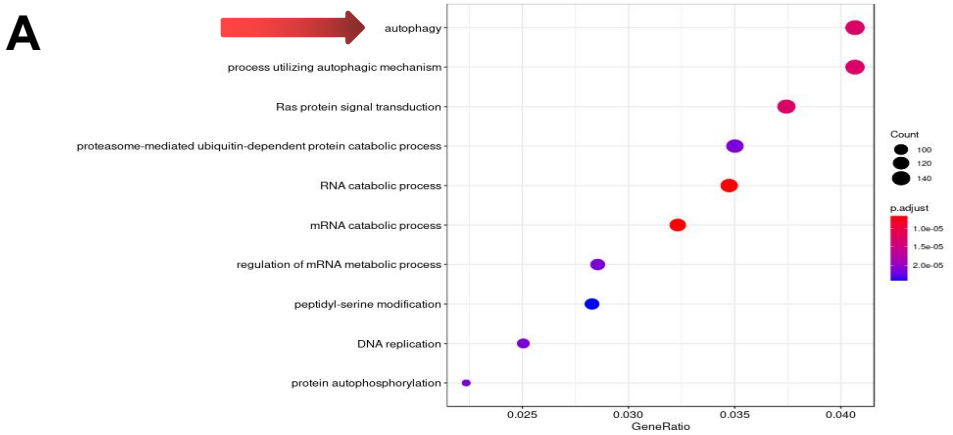

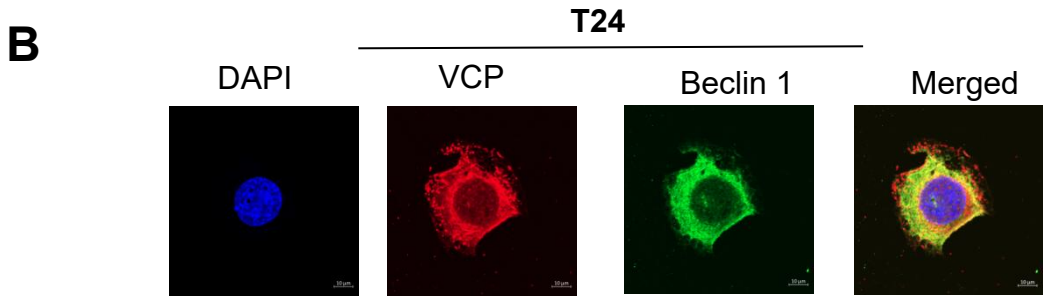

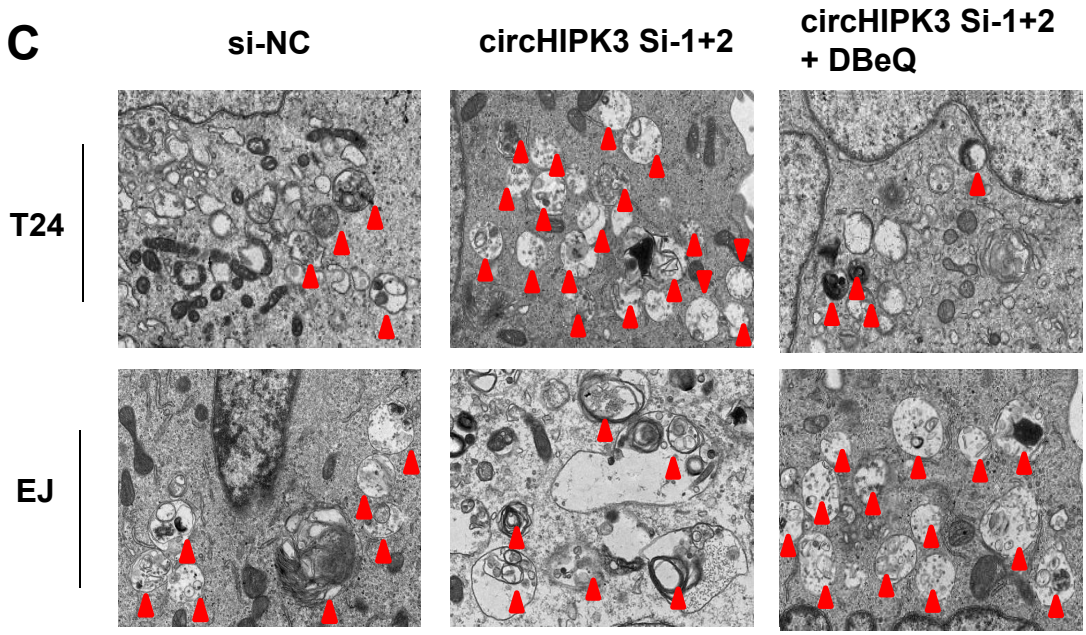

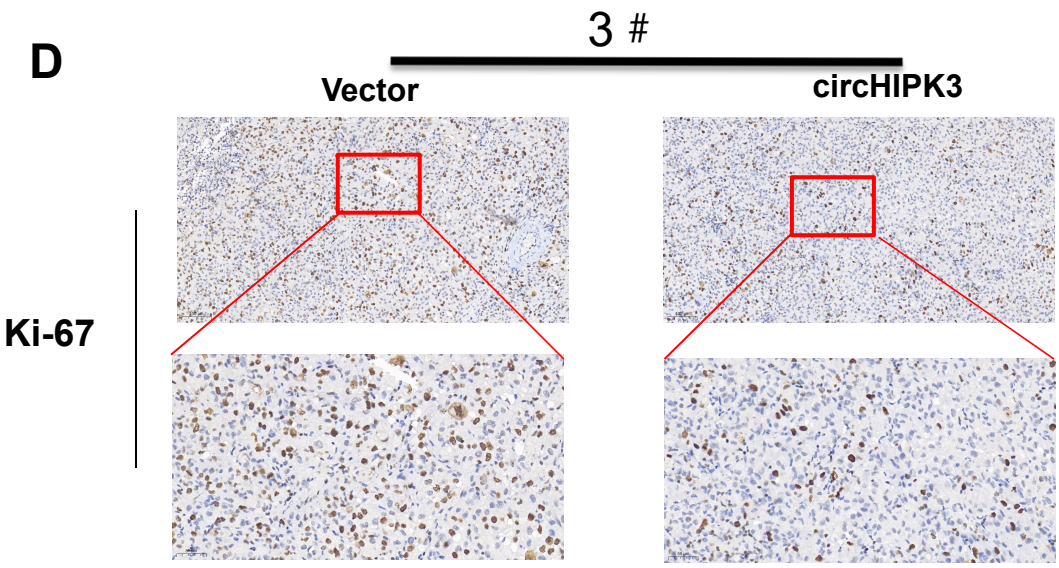

Supplemental Figure 4

A

Table 1

Tumor volume (mm^3)								
	Vector				circHIPK3			
0W	0	0	0	0	0	0	0	0
1W	0	0	0	0	0	0	0	0
2W	125.5	105.2	115.4	118.6	50.6	55.5	48.6	49.6
3W	259.6	210.4	230.6	225.5	90.5	99.8	89.9	90.8
4W	520	421.2	475.7	459.5	174.9	206.5	190.5	180.6

Table 2

Tumor weight (mg)		
	Vector	circHIPK3
1#	305.5	134.2
2#	224.2	100.4
3#	238.1	105.6
4#	203.2	99.8
