# Supplementary material for: CircHIPK3 negatively regulates autophagy by blocking VCP binding to the Beclin 1 complex in bladder cancer
Source: Discov Oncol. 2023 Jun 3;14:86. doi: 10.1007/s12672-023-00689-0 (PMC10239413; doi:10.1007/s12672-023-00689-0)

Figure 3

A

P62

Beclin 1

LC3B-Ⅰ
LC3B-Ⅱ

β-actin

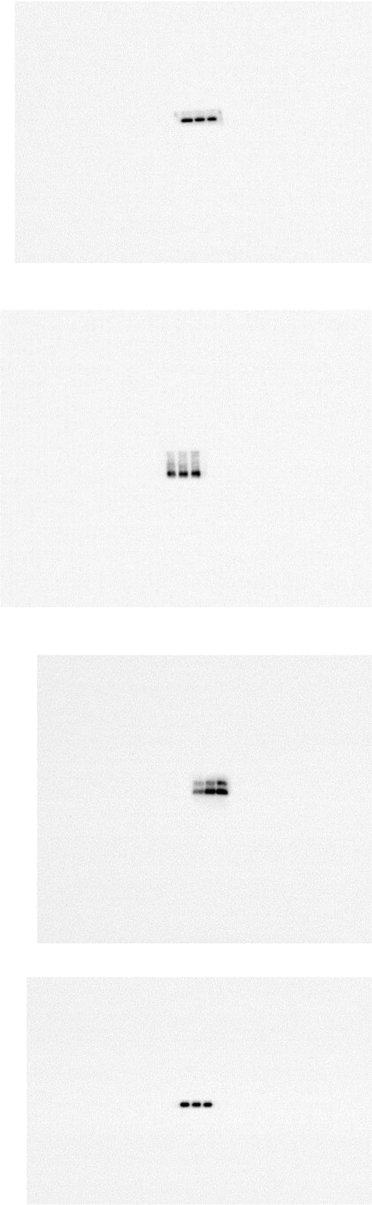

B

LC3B-Ⅰ
LC3B-Ⅱ

β-actin

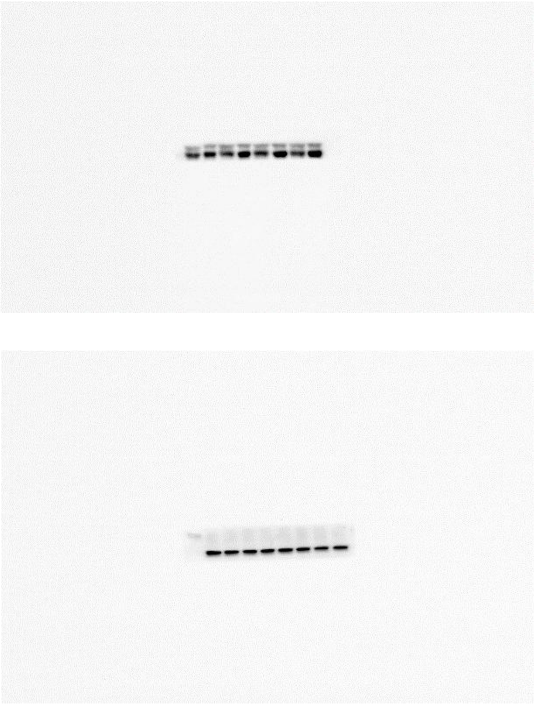

Figure 3

C

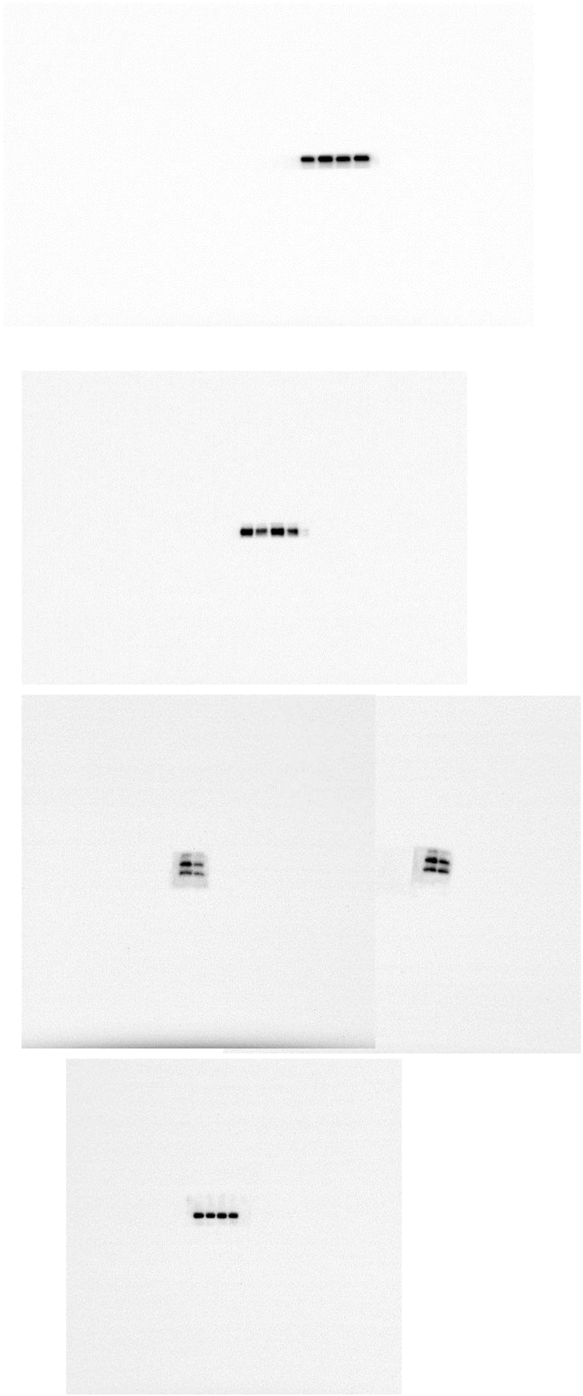

P62

Beclin 1

LC3B-Ⅰ
LC3B-Ⅱ

β-actin

Figure 3

D

P62

Beclin 1

LC3B-Ⅰ
LC3B-Ⅱ

β-actin

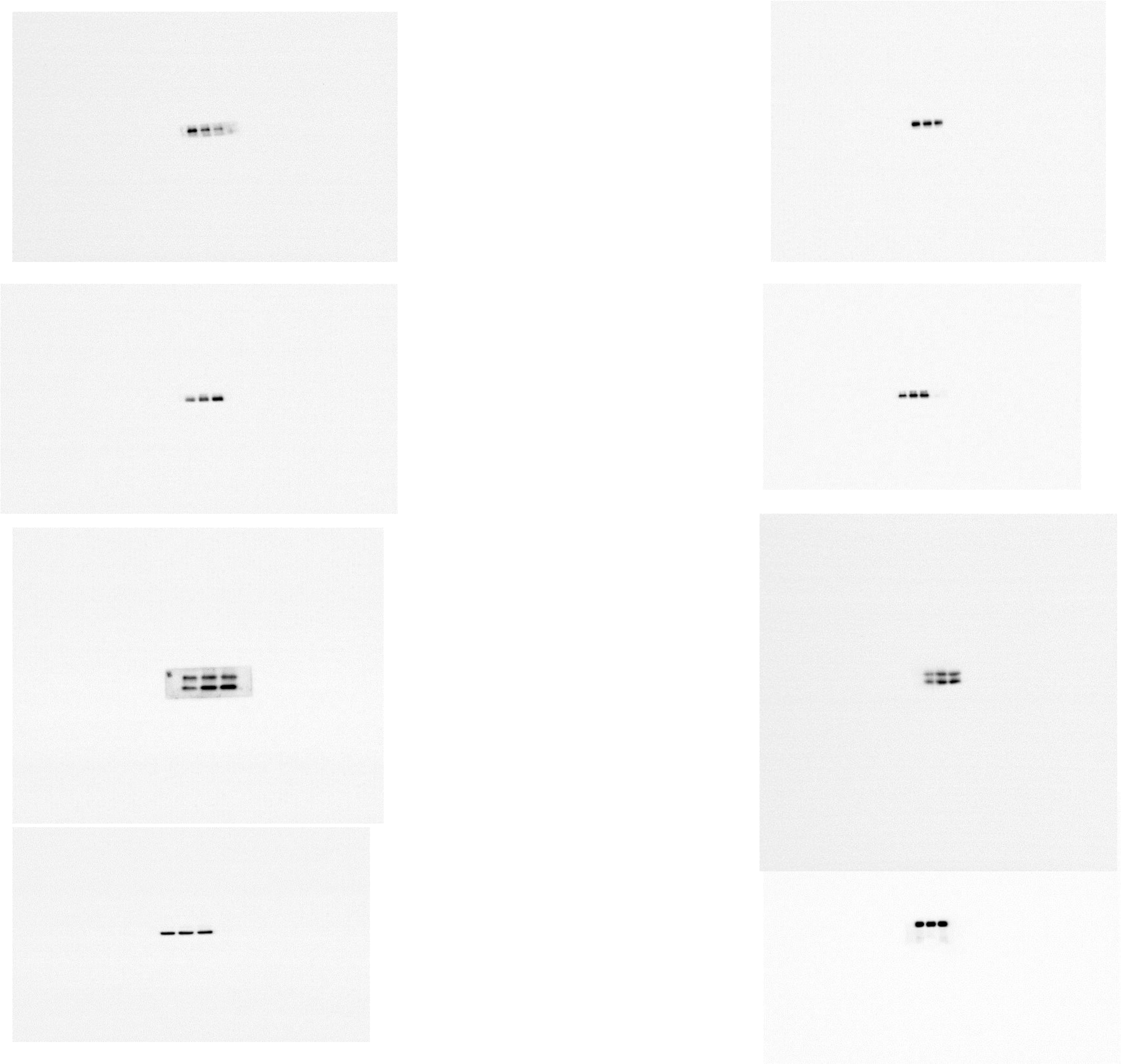

Figure 4

C

T24

VCP

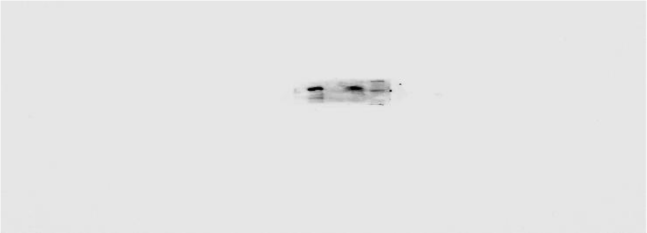

Beclin 1

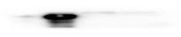

EJ

VCP

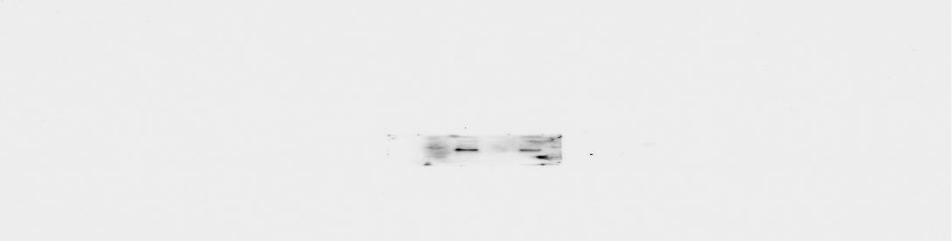

Beclin 1

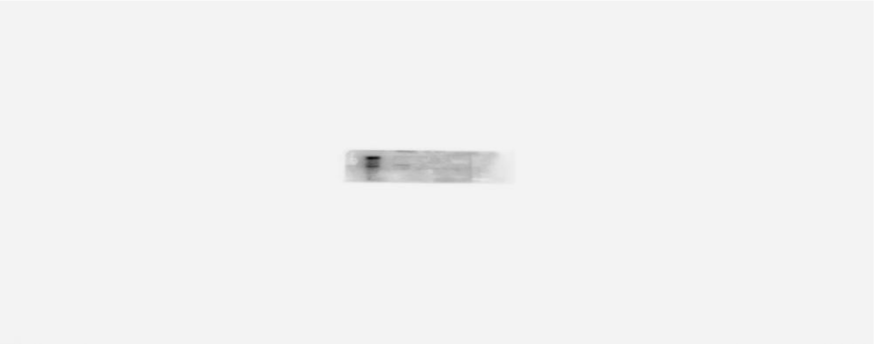

E

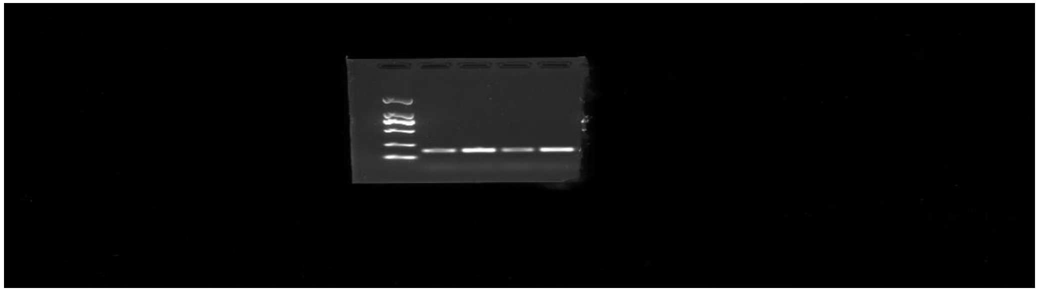

Figure 5

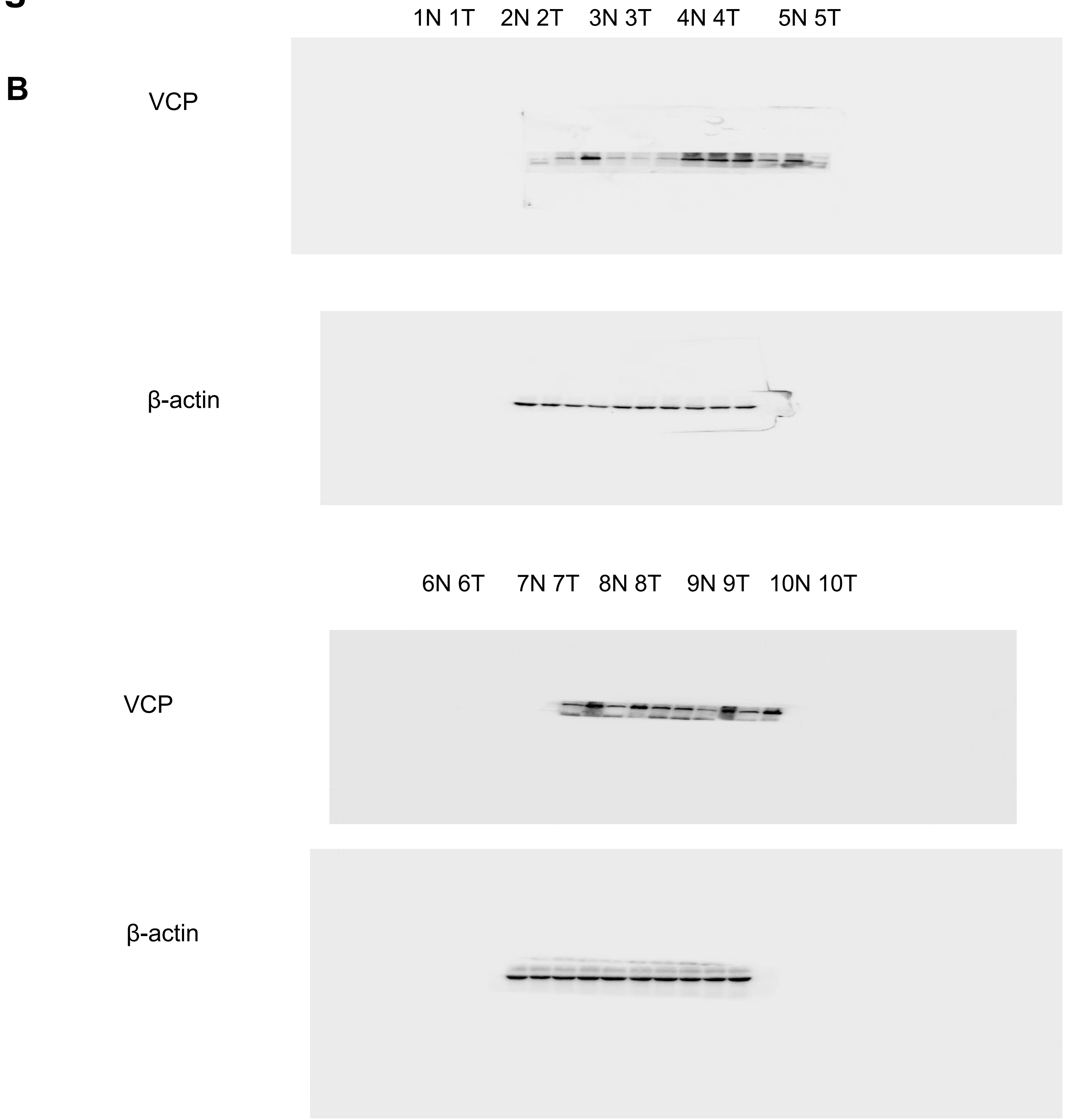

B
1N 1T 2N 2T 3N 3T 4N 4T 5N 5T
VCP
β-actin
6N 6T 7N 7T 8N 8T 9N 9T 10N 10T
VCP
β-actin

Figure 5

B

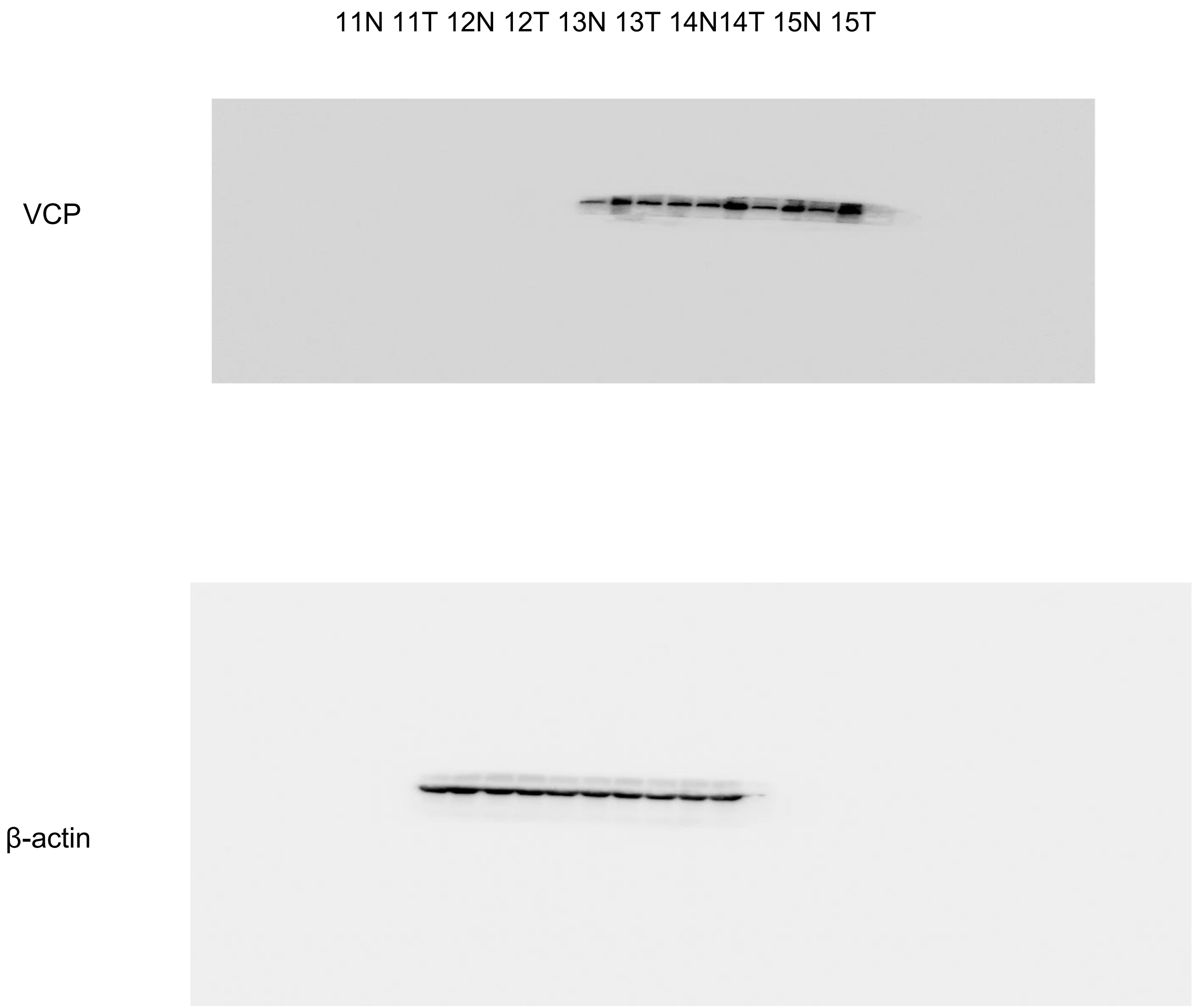

Figure 5

C

VCP

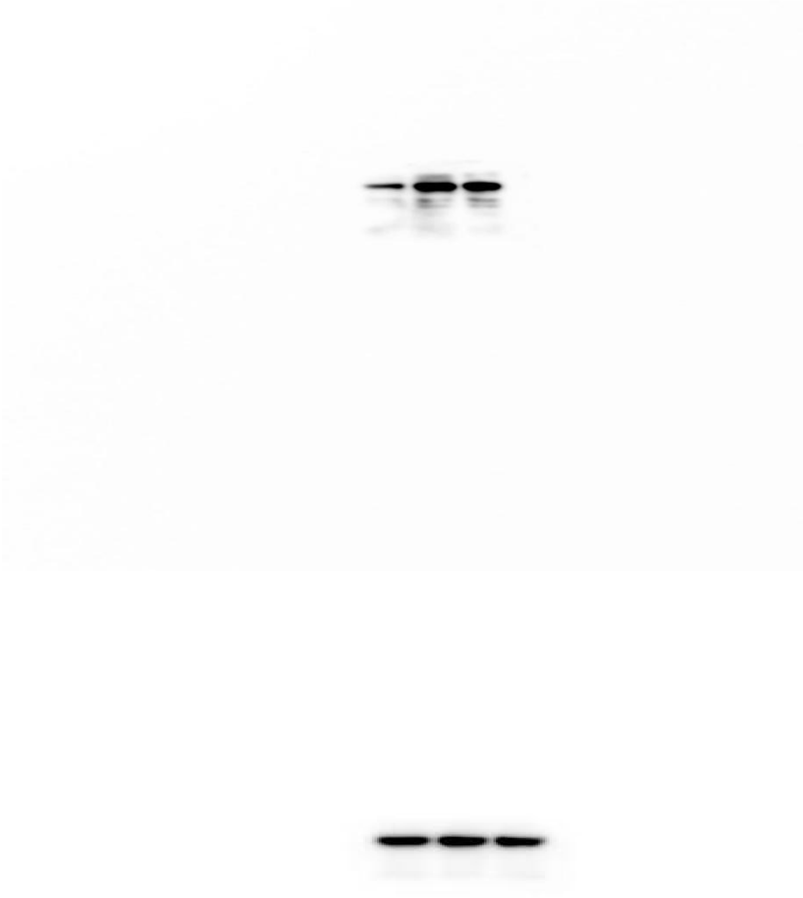

β-actin

Figure 5

D

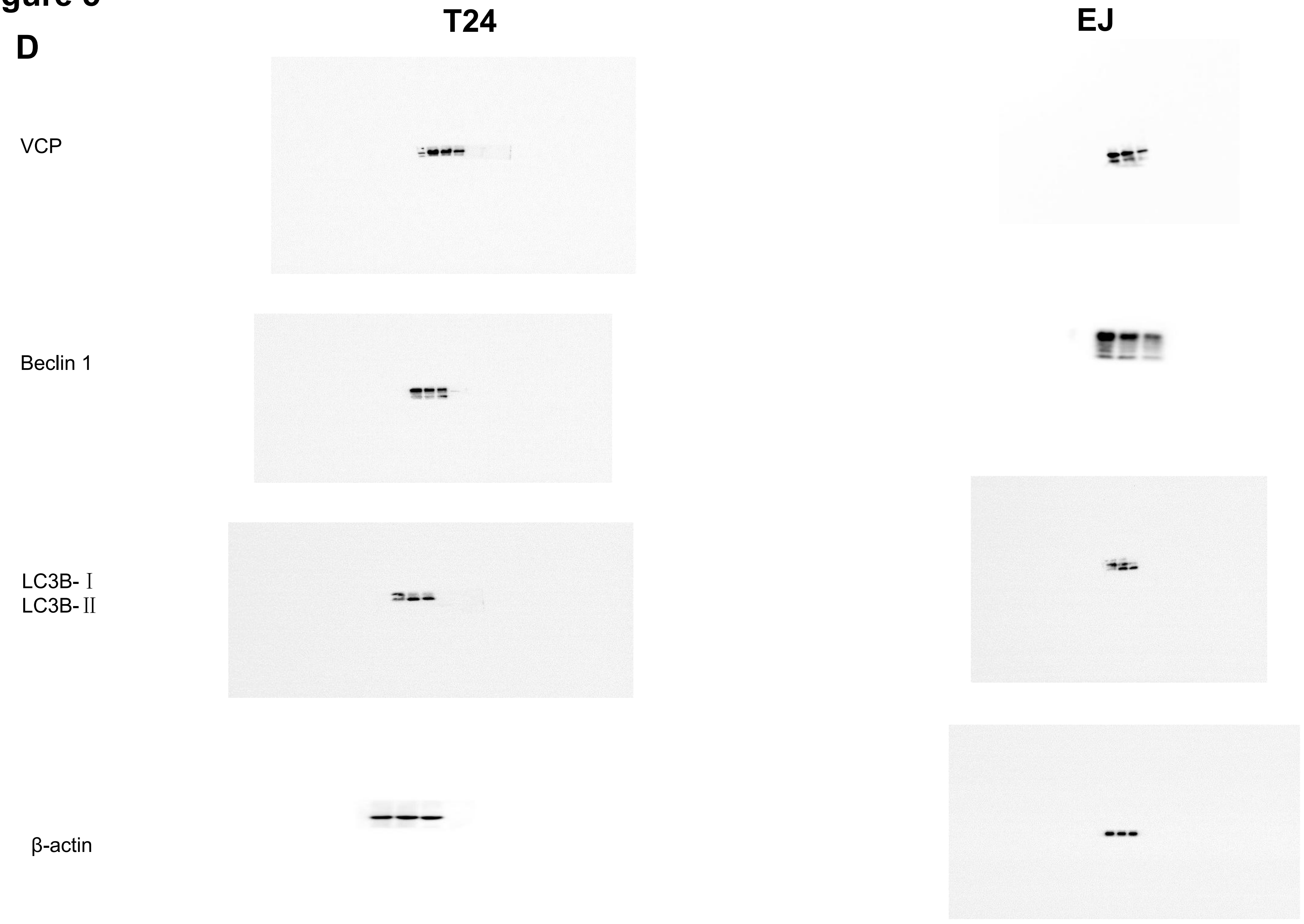

Figure 5

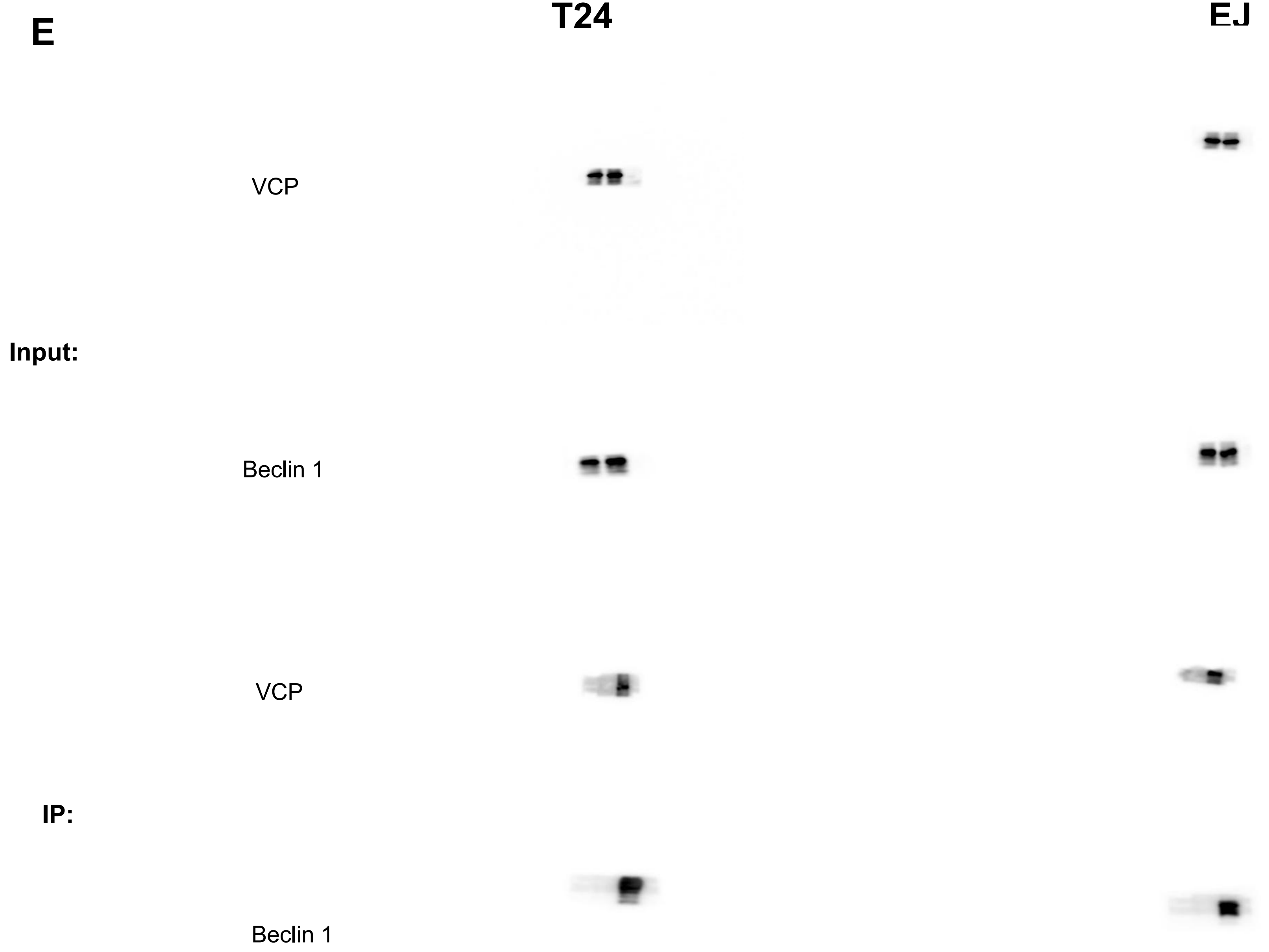

Figure 5

F

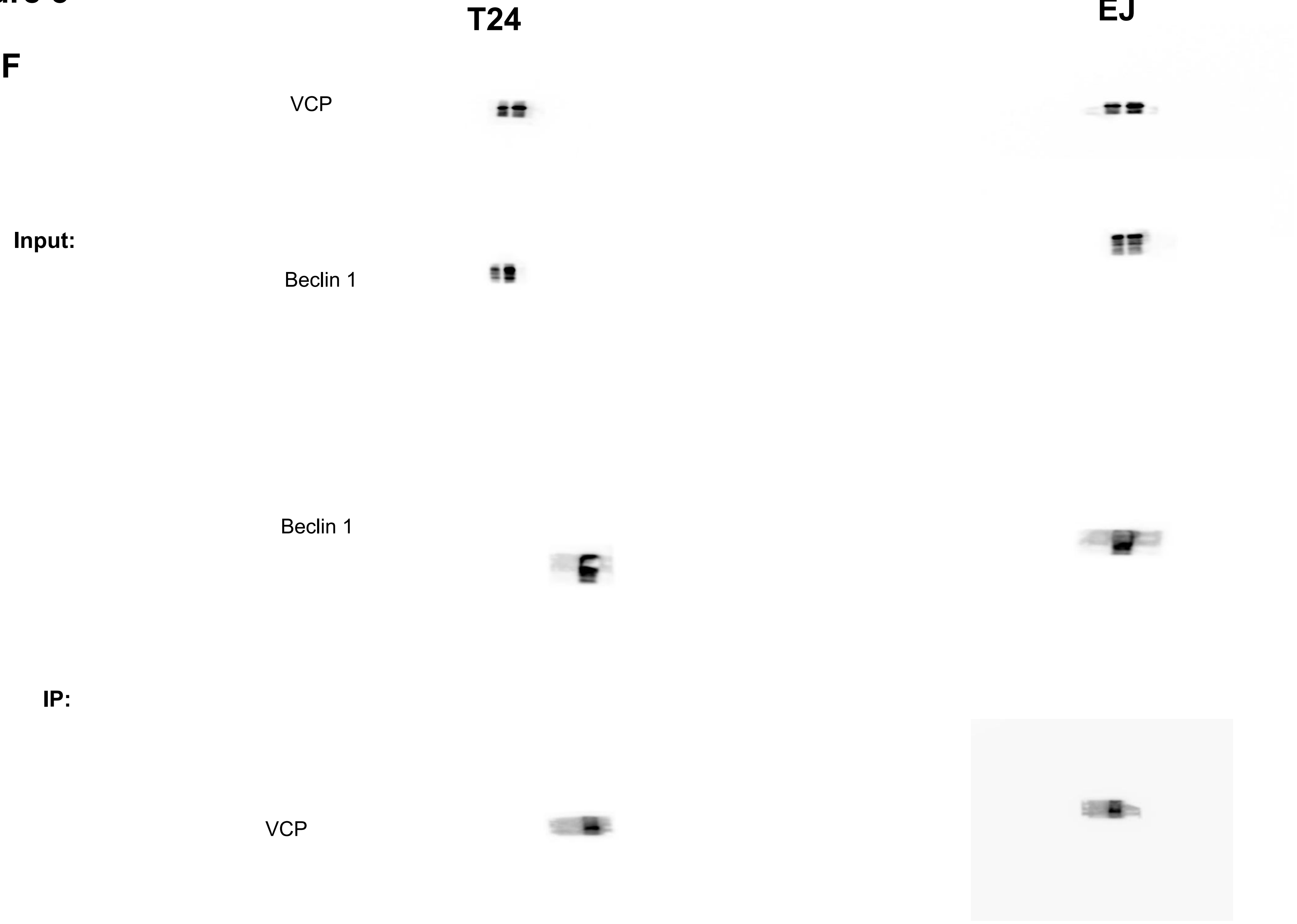

Figure 5

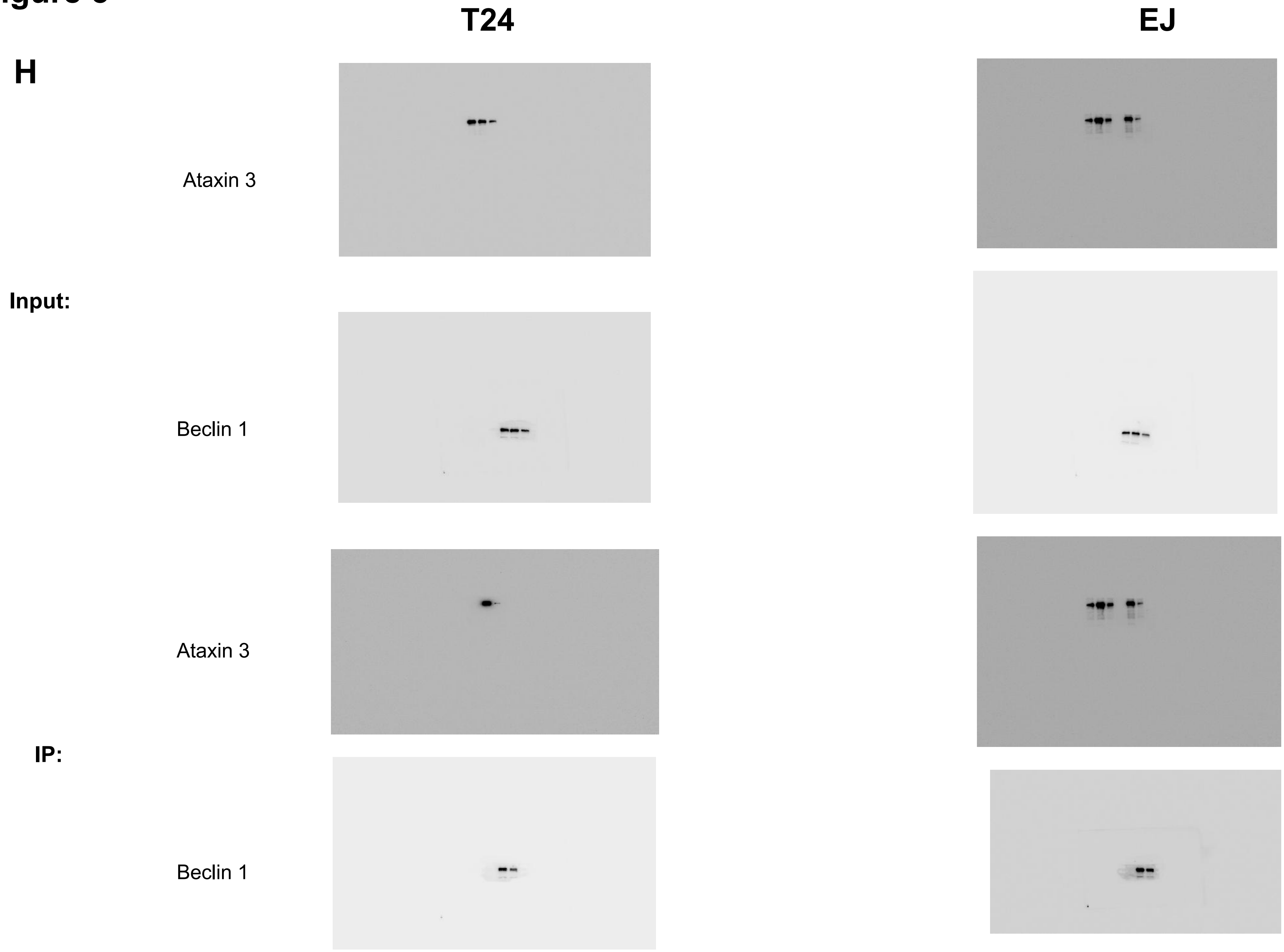

Figure 6

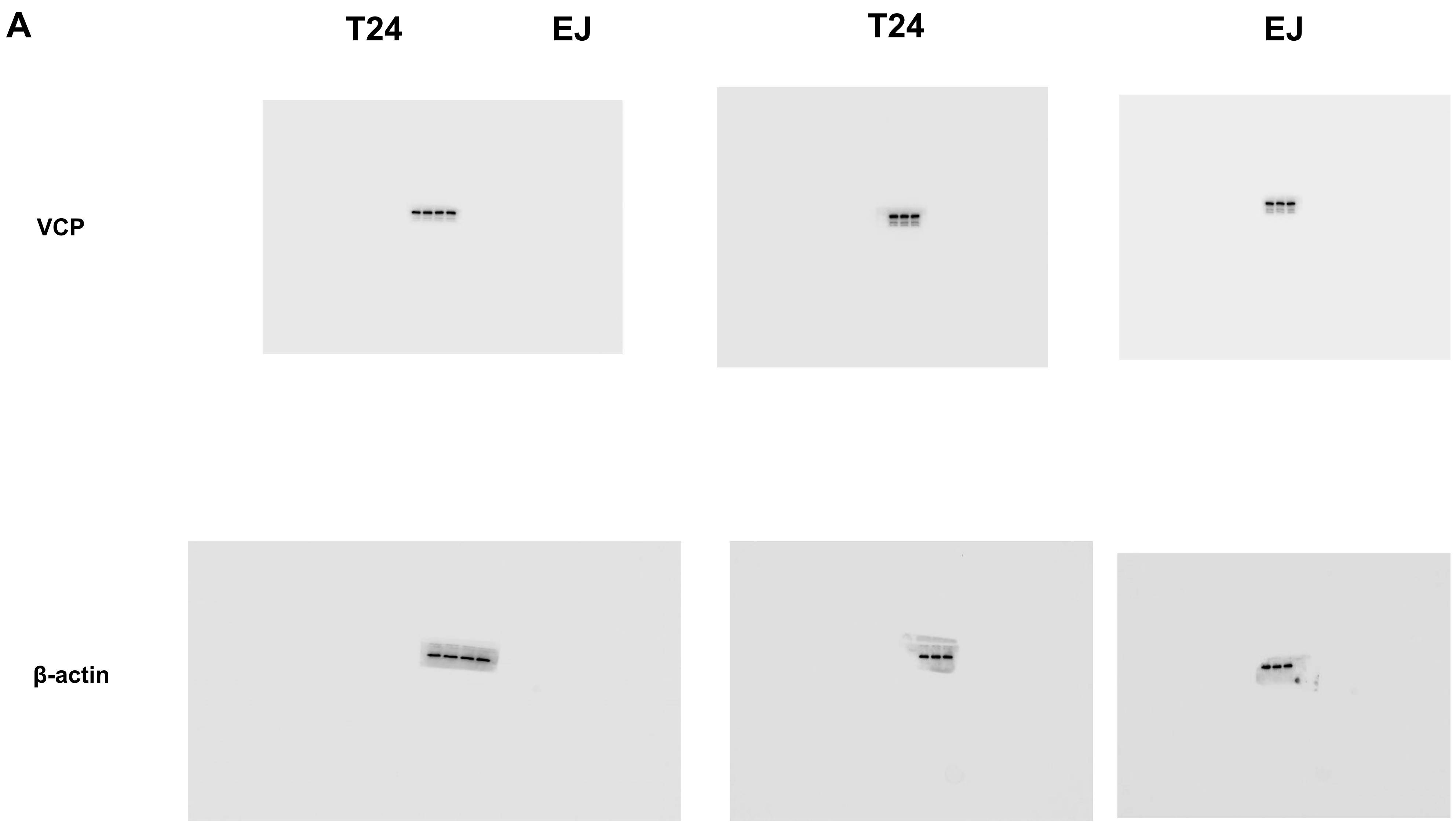

Figure 6

C

T24 **EJ**

Input:

VCP

Beclin 1

IP:

Beclin 1

VCP

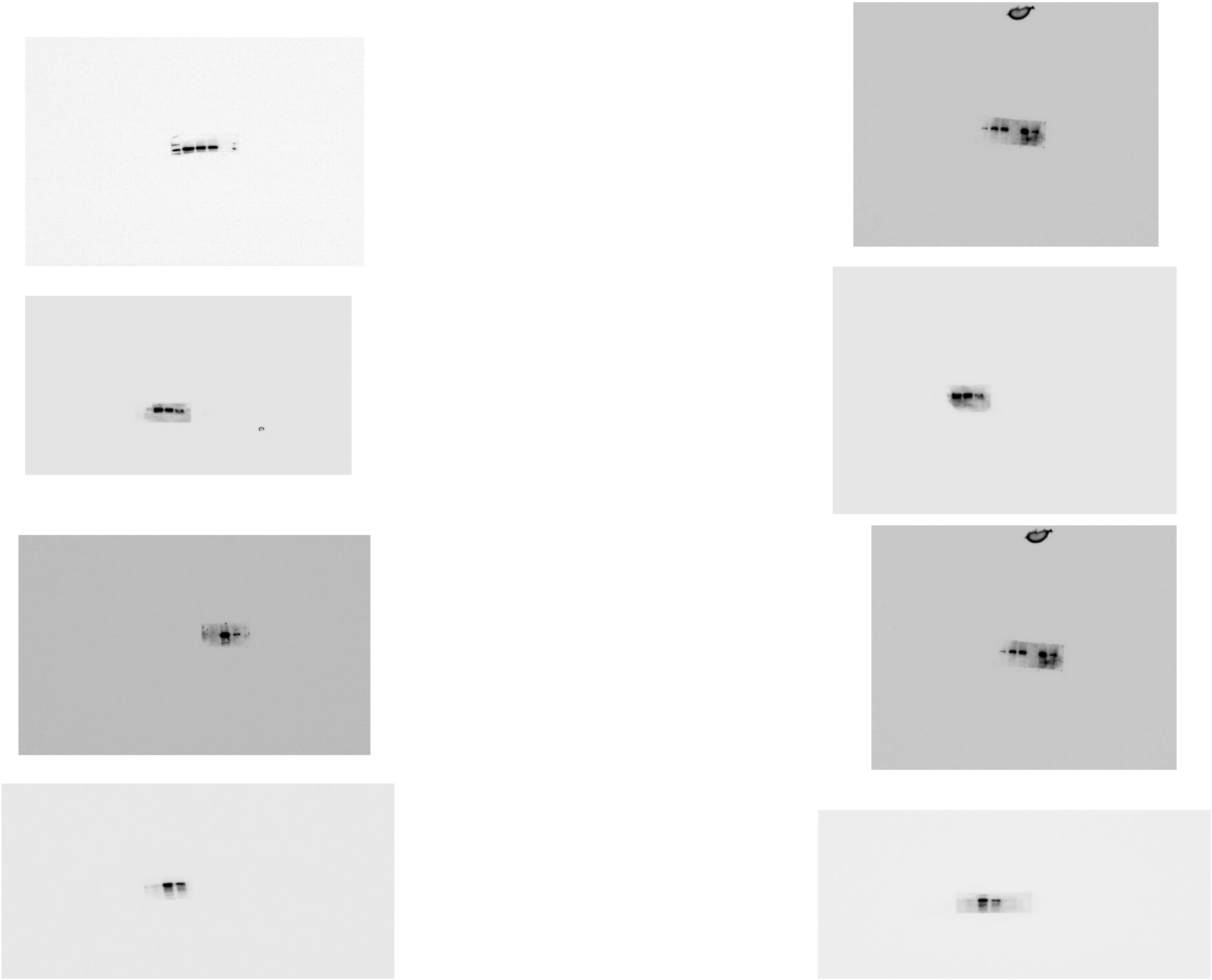

Figure 8

C

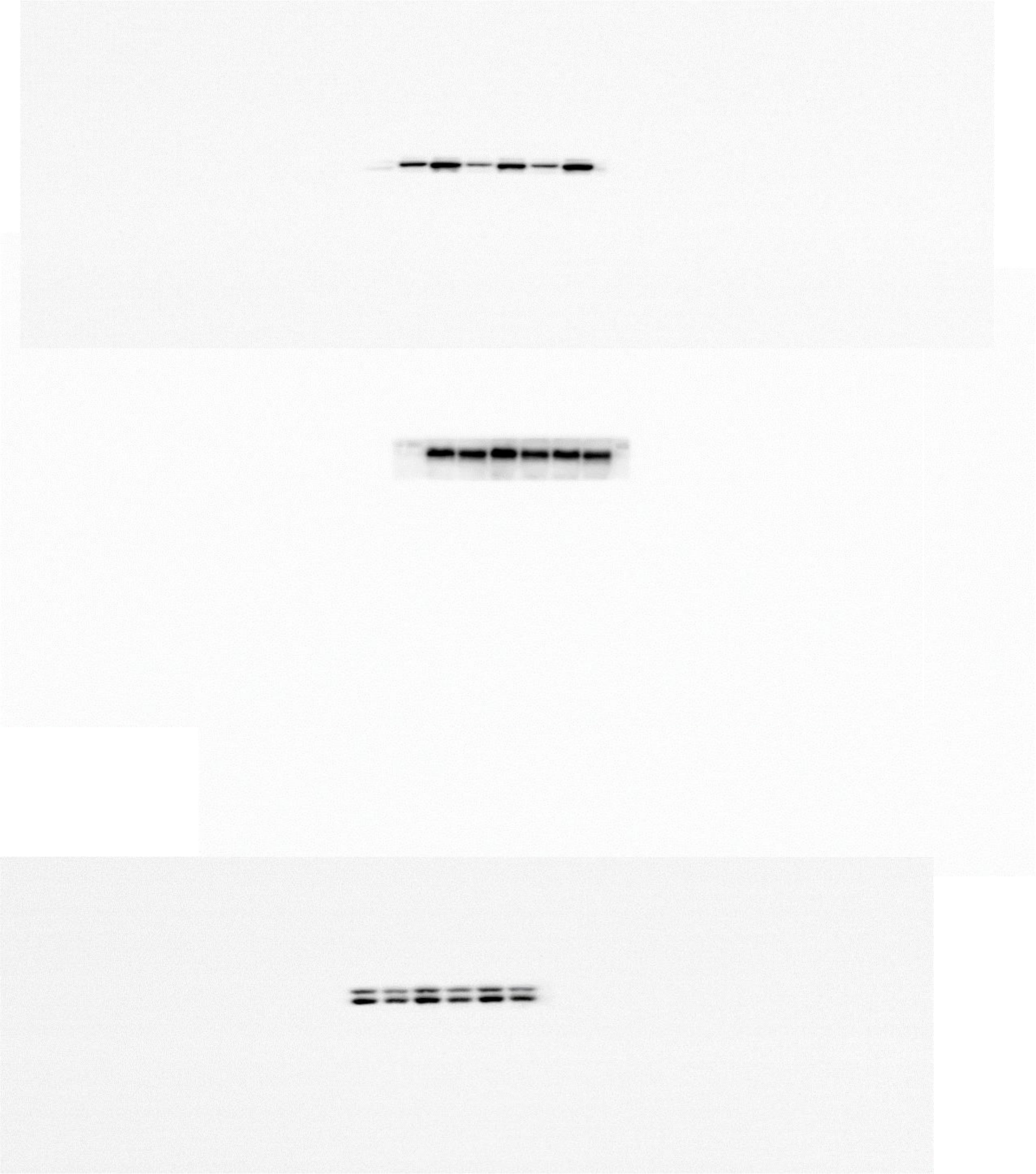

P62

Beclin 1

LC3B-Ⅰ
LC3B-Ⅱ

Figure 8

C

VCP

Ataxin 3

β-actin

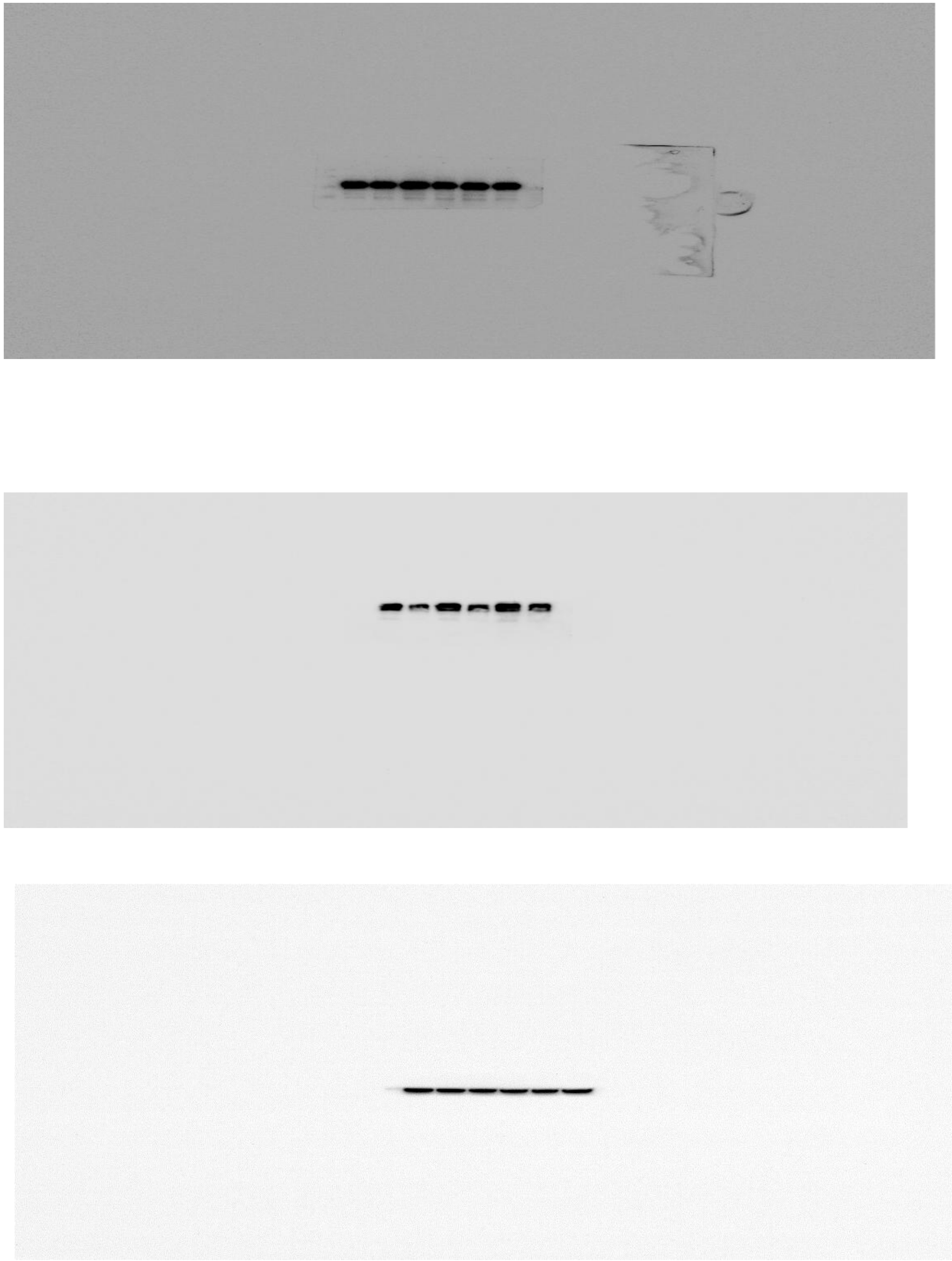

Supplement: Supplementary file 2 — Additional file 2. Western Blotting [file 12672_2023_689_MOESM2_ESM.pdf]
